# Edaravone and obeticholic acid protect against cisplatin-induced heart toxicity by suppressing oxidative stress and inflammation and modulating Nrf2, TLR4/p38MAPK, and JAK1/STAT3/NF-κB signals

**DOI:** 10.1007/s00210-024-02956-5

**Published:** 2024-01-29

**Authors:** Ehab A. M. El-Shoura, Emad H. M. Hassanein, Hesham H. Taha, Abdel-Gawad S. Shalkami, Mohamed Mahmoud Hussein Hassanein, Fares E. M. Ali, Adel G. Bakr

**Affiliations:** 1 Department of Clinical Pharmacy, Faculty of Pharmacy, Al-Azhar University, Assiut Branch, Assiut, Egypt; 2 Department of Pharmacology and Toxicology, Faculty of Pharmacy, Al-Azhar University, Assiut Branch, Assiut, 71524 Egypt; 3 Biochemistry and Molecular Biology Department, Faculty of Pharmacy, Al-Azhar University, Assiut Branch, Assiut, Egypt; 4 Clinical Pharmacy Program, Faculty of Health Science and Nursing, Al-Rayan Colleges, Medina, Kingdom of Saudi Arabia; 5 Forensic Medicine and Clinical Toxicology Department, Faculty of Medicine, Al-Azhar University, Assiut, Egypt

**Keywords:** Edaravone, Obeticholic acid, Cisplatin, Cardiotoxicity, Nrf2, JAK1/STAT3/NF-κB

## Abstract

Cardiotoxicity is a significant adverse effect of cisplatin (CIS) that necessitates extensive medical care. The current study examines the cardioprotective effects of edaravone (EDV), obeticholic acid (OCA), and their combinations on CIS-induced cardiac damage. Rats were allocated into five groups: the normal control group, the remaining four groups received CIS (7.5 mg/kg, i.p.) as a single dose on the fifth day and were assigned to CIS, OCA (10 mg/kg/day) + CIS, EDV (20 mg/kg/day) + CIS, and the (EDV + OCA) + CIS group. Compared to the CIS-treated group, co-treating rats with EDV, OCA, or their combinations significantly decreased ALP, AST, LDH, CK-MB, and troponin-I serum levels and alleviated histopathological heart abnormalities. Biochemically, EDV, OCA, and EDV plus OCA administration mitigated cardiac oxidative stress as indicated by a marked decrease in heart MDA content with a rise in cardiac antioxidants SOD and GSH associated with upregulating Nrf2, PPARγ, and SIRT1 expression. Besides, it dampened inflammation by decreasing cardiac levels of TNF-α, IL-1β, and IL-6, mediated by suppressing NF-κB, JAK1/STAT3, and TLR4/p38MAPK signal activation. Notably, rats co-administered with EDV plus OCA showed noticeable protection that exceeded that of EDV and OCA alone. In conclusion, our study provided that EDV, OCA, and their combinations effectively attenuated CIS-induced cardiac intoxication by activating Nrf2, PPARγ, and SIRT1 signals and downregulating NF-κB, JAK1/STAT3, and TLR4/p38MAPK signals.

**Graphical Abstract:**

Outlined diagram summarized the possible protective mechanisms of OCA and/or EDV against cisplatin-induced cardiac injury

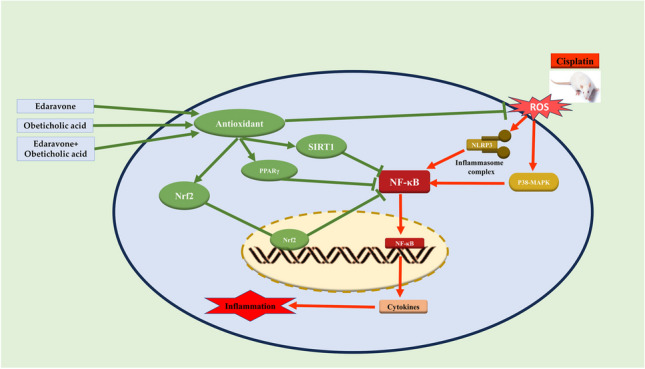

**Supplementary Information:**

The online version contains supplementary material available at 10.1007/s00210-024-02956-5.

## Introduction

Cisplatin (CIS) is a well-known chemotherapy drug used to remedy various cancers, such as breast, testicular, ovarian, and others (Ghosh 2019; Dasari and Tchounwou 2014). Nevertheless, the therapeutic use of CIS is restricted by multiple organ toxicities, including renal toxicity, cardiotoxicity, bone marrow suppression, and others (Qi et al. 2019). Notably, free radicals have been involved in CIS-induced multiple organ injury, as reactive oxygen species (ROS) can activate enormous output of proinflammatory cytokines such as tumor necrosis factor-alpha (TNF-α) and interleukin (IL)-1β and IL-6 (Miller et al. 2010; Barabas et al. 2008; El-Awady el et al. 2011). These proinflammatory cytokines and free radicals significantly harm the heart (El-Awady el et al. 2011). However, the exact mechanism of toxicity is still obscure.

Oxidative stress induces the alteration of different signaling pathways. The antioxidant response element (ARE) mediated by the activation of transcription factor nuclear factor erythroid 2–related factor 2 (Nrf2) protects the cells from the harmful effect of oxidative stress and potently mitigates the cytotoxic effect caused by diverse free radicals and inflammatory signals (Hassanein et al. 2020b; Loboda et al. 2016). In addition, a protein named silent information regulator T1 (SIRT1), an NAD + -dependent histone deacetylase, plays diverse roles in oxidative stress, apoptosis, inflammation, and aging (Singh and Ubaid 2020). Many investigations documented that the SIRT1 signal possesses a valuable function against cardiac damage and can represent a therapeutic target for inflammation-related problems (Packer 2020). Moreover, a nuclear receptor called peroxisome proliferation-activated receptor gamma (PPARγ) controls the transcription of several genes primarily involved in fatty acid and energy metabolism. The activation of PPARγ influences a wide range of biological processes, including the regulation of metabolism, the suppression of inflammation, the control of immune cell balance, the inhibition of apoptosis and oxidative stress, and the enhancement of endothelial function (Ivanova et al. 2015).

Studies indicate that most mechanisms behind the progression of structural heart disease include inflammation, and anti-inflammatory medications can decrease cardiovascular events because inflammation is a major contributing factor to cardiovascular disorders (Kalogeropoulos et al. 2012). Additionally, free radicals activate inflammatory signals such as stimulation of mitogen-activated protein kinases (MAPKs) and nuclear factor-*κappa* B (NF-κB) (Al-Kahtani et al. 2014). Besides, the Janus kinase/signal transducers and activators of transcription (JAK/STAT) signal are an intracellular signal transduction pathway ubiquitously expressed and involved in numerous biological functions. It offers a direct mechanism for regulating gene expression by external stimuli. Strong evidence suggests several inflammatory and immunological disorders are intimately linked to the prolonged and excess activation of the JAK/STAT signaling pathway (Xin et al. 2020). Side by side, the innate inflammatory process is significantly influenced by toll-like receptors (TLR). TLR4 is emerging as the most well-known of TLR members (Moresco et al. 2011). TLR4 stimulation triggered the inflammatory cascade and mediated inflammatory responses through the crucial downstream effector NF-κB (Dou et al. 2013).

Obeticholic acid (OCA), a semisynthetic derivative of chenodeoxycholic acid, is selective and potent farnesoid X receptor (FXR) agonist (Abenavoli et al. 2020). Many investigations reported that FXR receptors are important in regulating cardiovascular biology (Li et al. 2020). OCA possesses antioxidant and anti-inflammatory effects in different models (Guo et al. 2021; Pellicciari et al. 2002). Edaravone (EDV) is a potent free radical scavenger used for amyotrophic lateral sclerosis (Watanabe et al. 2008; Rothstein 2017). Previous studies reported that EDV has promising protective effects in different animal models, such as myocardial injury (Iguchi et al. 2004), CIS-activated acute renal toxicity (Zhang et al. 2003), and others. As mentioned before, the scope of this work was to inspect the potential protective effect of EDV and OCA against CIS-induced cardiac injury in rats, pointing out the role of Nrf2, PPARγ, SIRT1, NF-κB, JAK/STAT, and TLR4/p38MAPK signals.

## Materials and methods

### Reagents and chemicals

Cisplatin MYLAN® (50 mg/50 ml) vial was used in the present study: EDV and OCA (Sigma Aldrich, St. Louis, MO, USA). All the remaining chemicals and reagents were of analytical category and obtained from standard mercantile provisions.

### Animals

The ethical use of laboratory animals was carried out according to the National Institute of Health (NIH) Guide for Care and Use of Laboratory Animals and approved from the Faculty of Pharmacy Ethical Committee et al.-Azhar University (approval number: ZA-AS/PH/5/C/2022). Forty male Wistar rats (180–200 g) were used in this work. Rats were acclimatized for 1 week before the experiments, under standard laboratory conditions, free water, and diet under a temperature of 25 ± 2 °C and humidity of 60 ± 10% and 12-h light and dark cycles.

### Experimental groups and treatment protocol

**Fig. 1:**
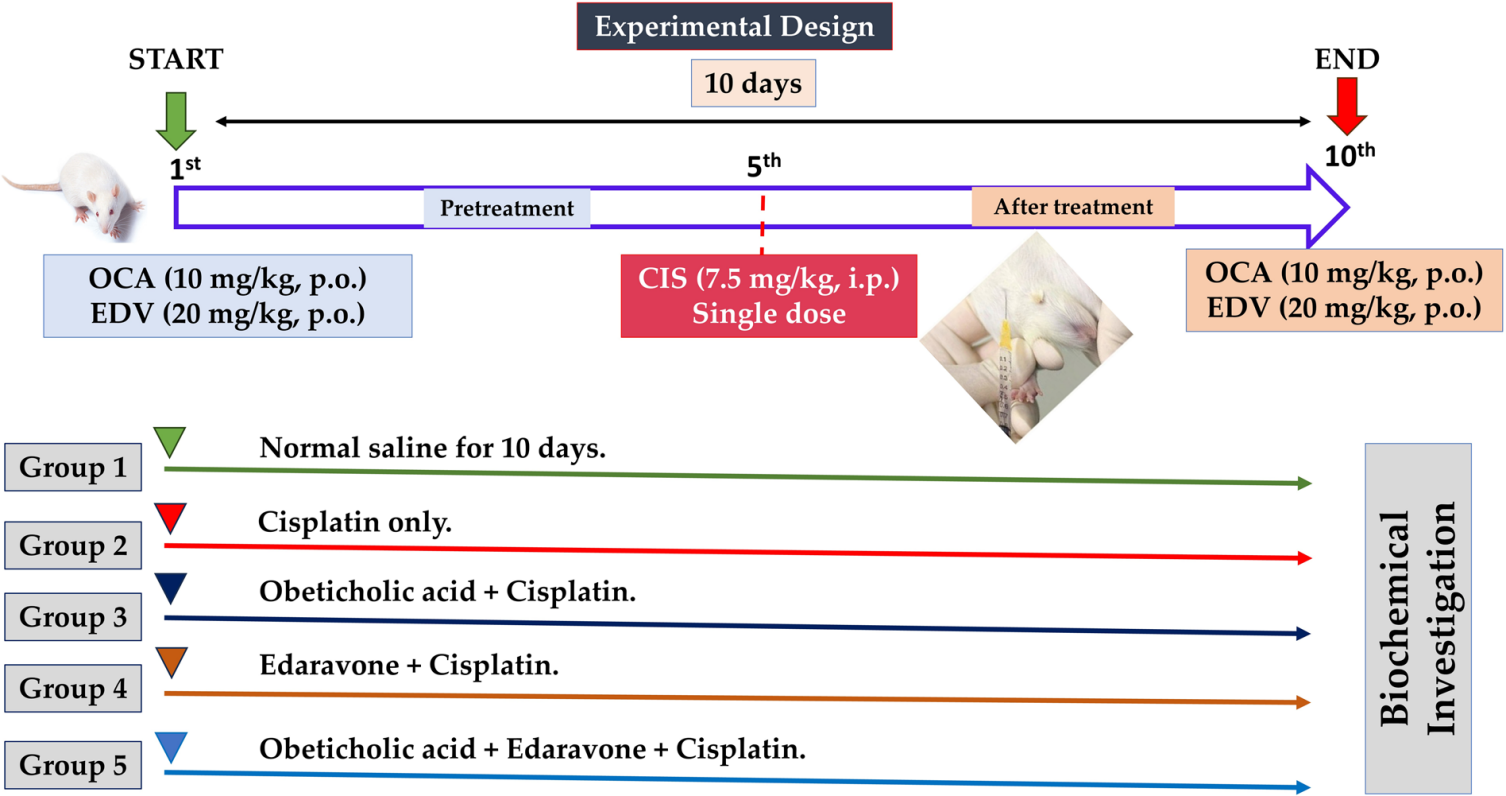
Illustrated diagram explored the study experimental design

After determining the effective dose of CIS, EDV, and OCA, the experimental design of the study has been summarized as follows (Fig. 1):

In brief, the animals were assigned into five groups (*n* = 8/group):

- Group 1: Normal control received saline daily for 10 days.
- Group 2: Received CIS (7.5 mg/kg, i.p.) single dose (Sherif 2021) on the 5th day of the study.
- Group 3: Received OCA (10 mg/kg, orally) (Ferrigno et al. 2020) for 10 successive days plus CIS (7.5 mg/kg, i.p.) on the 5th day of this study.
- Group 4: Received EDV (20 mg/kg, orally) (Hassanein et al. 2020a) for 10 successive days plus CIS (7.5 mg/kg, i.p.) (Emekli-Alturfan et al. 2015) on the 5th day of this study.
- Group 5: Received OCA + EDV for 10 successive days at doses mentioned before and CIS on the 5th day of this study.

### Sample collection and tissue processing

After 10 days of the treatment protocol, blood was obtained from the retroorbital plexus and under ketamine (100 mg/kg, i.p.) anesthesia and allowed to clot for serum preparations obtained by centrifugation at 1200 g for 15 min for the biochemical markers. Then, rats were sacrificed, and hearts were dissected and washed with ice-cold saline. A small piece of cardiac tissue from each group was preserved in 10% neutral formalin for histopathological evaluation. A large piece of cardiac tissues was divided into two parts; one section was homogenized in phosphate buffer at pH 7.4 to produce cardiac homogenates for oxidative stress biomarkers spectrophotometrically and cytokine assessment by enzyme-linked immunosorbent assay (ELISA). Other cardiac tissue sections were stored in RNAlater for quantitative real-time polymerase chain reaction (q-RCR) estimation and lysis buffer for western blotting evaluation.

### Determination of serum ALP, AST, LDH, CK-MB, and troponin-I levels

Rats’ sera were used for the measurement of alkaline phosphatase (ALP; CAT# 1,001,132; Spinreact, Spain), aspartate aminotransferase (AST; CAT# 1,001,162; Spinreact, Spain), lactate dehydrogenase (LDH; CAT# 1,001,260; Spinreact, Spain), creatinine kinase-MB (CK-MB; CAT# 41,254; Spinreact, Spain), and troponin-I (CAT# E-EL-R1253; Elabscience, China) levels according to the manufacturer’s instructions.

### Histological examinations

Cardiac sections were settled in 10% neutral formalin and then desiccated, cleared, embedded in paraffin, cut into 4–5-µm-thick sections, and stained with hematoxylin and eosin (H&E) based on instructions notarized by Feldman and Wolfe (2014). The slides were evaluated randomly using a light microscope (Olympus, USA). On a scale of 0 to 4, the parameters of the pathological changes were tabulated and graded, with 0 signifying a healthy myocardium or the absence of the parameters in the samples: (1) showing the parameter’s observed presence and distribution up to 25% of the region under examination (0–25%); (2) observed existence and distribution of the parameter in between 26 and 50% of the area under study (26–50%); (3) observed existence and distribution of the parameter in between 51 and 75% of the area under study (51–75%); (4) showing that the parameter was present and distributed in more than 75% of the area under study (76–100%) (Molh et al. 2008).

### Estimation of antioxidant and oxidative stress parameters

To examine the cardiac oxidative stress, the activity of superoxide dismutase enzyme (SOD), contents of lipid peroxidation (MDA), and reduced glutathione (GSH) in the homogenates of cardiac tissues were measured by the method documented earlier by Marklund (1985), Mihara and Uchiyama (1978), Sedlak and Lindsay (1968), respectively. Also, the enzyme activity of myeloperoxidase (MPO) was estimated colorimetrically based on the instructions authenticated by Manktelow and Meyer (1986).

### Determination of inflammatory mediators

To examine CIS-induced inflammation in cardiac tissues, TNF-α (CAT# E-EL-R2856), IL-1β (CAT# E-EL-R0012), and IL-6 (CAT# E-EL-R0015) levels were measured by ELISA technique based on the manufacturer’s directions. The kits were purchased from Elabscience, China.

### Estimation of mRNA expressions by qRT-PCR

To inspect the impact of ED and OCA, mRNA expression of p38MAPK, TLR4, NF-κB-p65, and NLR family pyrin domain-containing 3 (NLRP3), genes were quantified by real-time PCR using the instrument, real-time PCR system (Applied Biosystems, USA). Briefly, total RNA was segregated by TRIzol reagent (Invitrogen, USA). The obtained RNA was determined by nanodrop and then used for cDNA synthesis. The resultant cDNA was aggrandized via SYBR green. The data obtained were evaluated using the 2^−ΔΔCt^ equation (Livak and Schmittgen 2001) and referenced GAPDH as a housekeeping gene. The primers are indexed in Table 1.

**Table 1:** Primer list for qRT-PCR

Target gene	The nucleotide sequence (5′‐3′)	Accession number	Product size
P38-MAPK	F: AGAGTCTCTGTCGACCTGCT R: CCTGCTTTCAAAGGACTGGT	XM_017601781.1	156
TLR-4	F: CGAGCCAGAATGAGGACTGG R: TCCCACTCGAGGTAGGTGTT	NM_019178.1	352
NLRP-3	F: GTGGCTACTCCCAGTGATTTGT R: TGCTTGCTTGGATGCTCCTT	XM_017597078.1	910
GAPDH	F: TGCTGGTGCTGAGTATGTCG R: TTGAGAGCAATGCCAGCC	NM_017008.4	645

### Western blotting

A small section of cardiac tissues was lysed using RIPA buffer, including protease inhibitor cocktail (Biospes, China), and centrifuged at (1200 g for 15 min at 4 °C). Total proteins were estimated from the resultant supernatant based on the method described by Bradford (1976). The proteins were loaded (50 µg in each lane) and then electrophoresed on SDS–polyacrylamide gels and relocated to a polyvinylidene difluoride membrane (Thermo Fisher Scientific, USA) through a semi-dry transfer manner (Towbin et al. 1979). After being blocked with 5% non-fat dried milk powder for 2 h, the membrane was mixed with primary antibodies overnight at 4°C (El-Shoura et al. 2018). Then, the membranes were handled with secondary antibodies at 37°C for 1 h. Finally, the bands were detected via 5-bromo-4-chloro-3-indolyl phosphate (BCIP)/nitro blue tetrazolium (NBT) system (Genemed, USA). The intensity of the bands was analyzed using ImageJ® software, and the values of each band were represented as the relative protein expression referenced to β-actin bands.

### Statistical analysis

Data are introduced by mean ± standard error of the mean. The data were tested for normality through the Shapiro–Wilk test. Multiple statistical comparisons were made via one-way ANOVA, followed by Tukey’s post-hoc test. Meanwhile, the Kruskal–Wallis test was employed to ascertain the difference between the groups obtained as semiquantitative in the histopathological examination. The level of significance at *P-*value < 0.05 was conceived as statistically significant. Differences were made between groups through the GraphPad Prism software (version 8.0, USA).

## Results

### Effect of OCA or EDV and their combination on serum ALP, AST, LDH, CK-MB, and troponin-I levels after CIS challenge

**Fig. 2:**
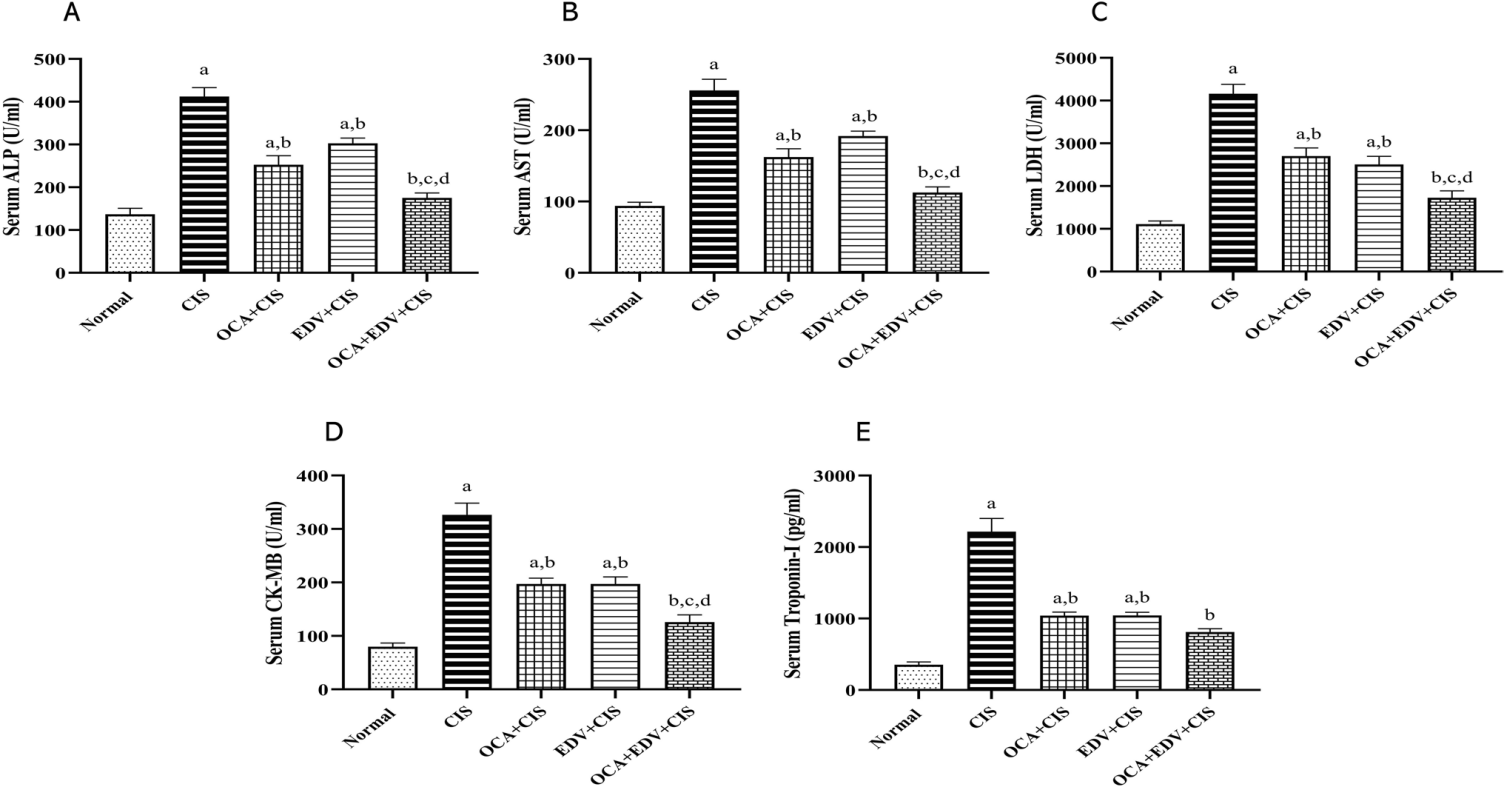
Effect of EDV, OCA, and their combination on serum ALP (**A**), AST (**B**), LDH (**C**), CK-MB (**D**), and troponin-I (**E**) levels after CIS challenge. Results are represented as mean ± SE of 8 independent values and statistically conducted using a one-way ANOVA test at *P*-value < 0.05. a represents a significant difference from the normal control group. b represents a significant difference from the CIS control group. c represents a significant difference from the OCA + CIS group. d represents a significant difference from the EDV + CIS group

Figure 2 shows that the serum ALP, AST, LDH, CK-MB, and troponin-I levels of rats in the CIS group were significantly increased compared with the normal control group. In contrast, co-treating rats with OCA or EDVs or their combinations potently decreased the serum levels of ALP, AST, LDH, CK-MB, and troponin-I. Notably, rats co-administered OCA plus EDV showed noticeably decreased ALP, AST, LDH, CK-MB, and troponin-I levels that exceeded that of OCA and EDV alone.

### Effect of OCA or EDV and their combination on histopathological abnormalities in CIS-injected rats

**Fig. 3:**
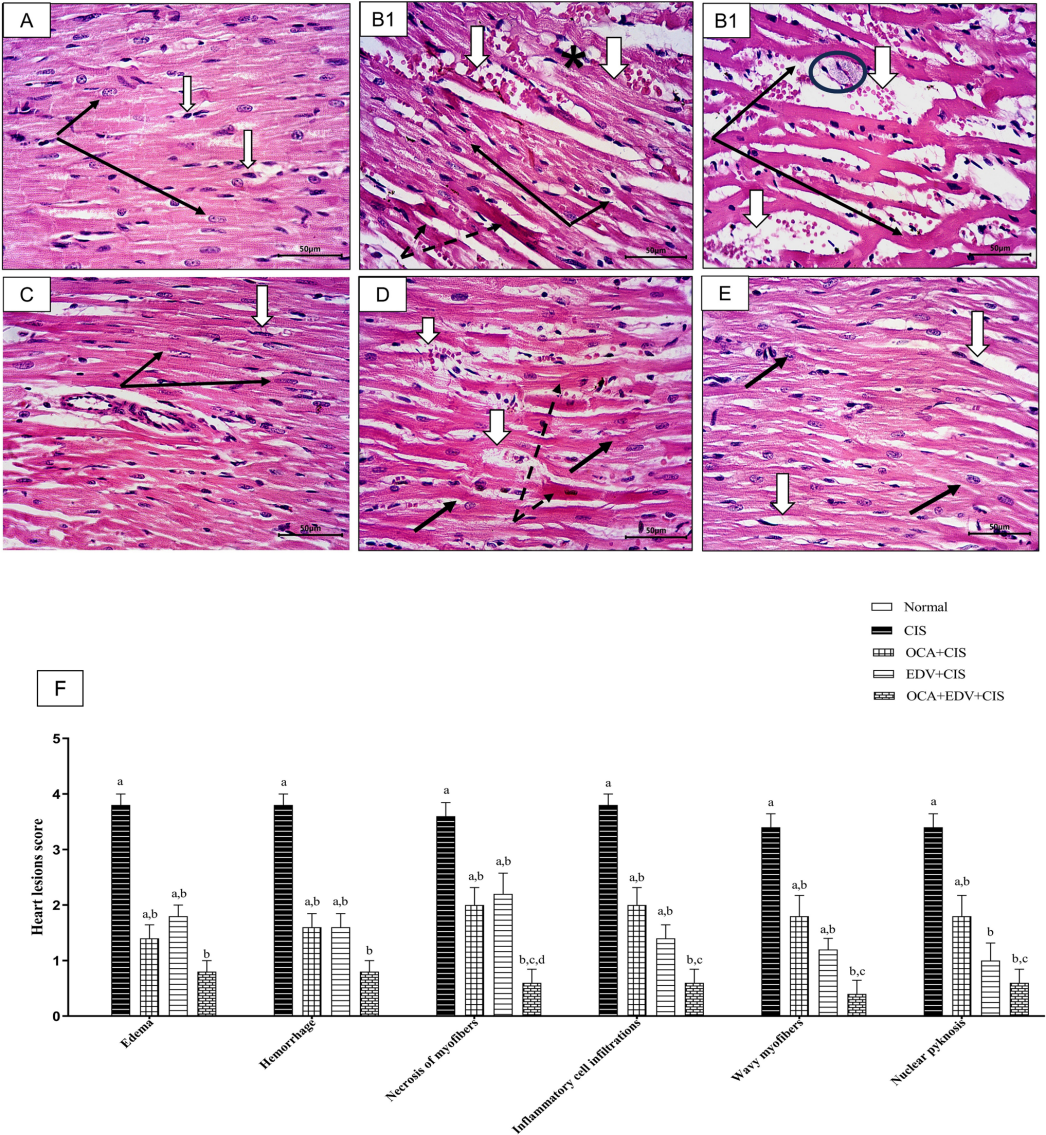
Effect of OCA or EDV and their combination on histopathological abnormalities in CIS-injected rats. **A** Sections from the left ventricle of the normal rat stained by H&E showed a regular pattern of cardiac muscle fibers with their oval active nuclei and fine transverse striation (black arrows). Blood capillaries are thin walls compressed among the muscles (white arrow). **B1** and **B2** Sections from CIS-administrated rats showed disorganization of normal patterns with signs of apoptosis represented by deeply eosinophilic stained fibers (dotted arrows) and pyknotic nuclei (black arrows). Sever edema (circle) and wavy myofibers (star) were also observed. Severely affected fibers showed thinning (black arrows) and interfibrillar hemorrhage (white arrows). **C** Sections from OCA plus CIS-administrated rats showed marked preservation of normal pattern arrangement and histological features of cardiac fibers (black arrows) with the absence of capillary congestion (white arrow) could be observed. **D** Sections from EDV plus CIS-administrated rats showed minor improvement where focal apoptotic degeneration is still observed (dotted arrows). Other fibers appeared thinner but with normal nuclei (black arrows). There is still capillary dilation (white arrows). **E** Sections from OCA + EDV plus CIS-administrated rats showed marked protection, and cardiac fibers pattern looked more or less similar to the normal control group. **F** Semiquantitative analysis of histopathological lesions in heart muscle. a represents a significant difference from the normal control group. b represents a significant difference from the CIS control group. c represents a significant difference from the OCA + CIS group. d represents a significant difference from the EDV + CIS group

Histological examination of cardiac tissue of rats stained by H&E showed that CIS administration resulted in disorganization of the normal pattern of cardiac muscles with signs of apoptosis where the muscles are stained deeply eosinophilic and showed pyknotic nuclei. In addition, severely affected fibers have been demonstrated in thinning and interfibrillar hemorrhage. In contrast, OCA treatment showed remarkable preservation of normal pattern arrangement and the histological features of cardiac muscles, while the treatment with EDV provided mild improvement of CIS-induced changes, and still, cardiac muscles appeared thinner but with normal nuclei. Besides, there is still capillary dilation. Interestingly, combination therapy between OCA plus EDV showed marked protection, and the cardiac fiber pattern looked more or less similar to the control group (Fig. 3).

### Effect of OCA or EDV and their combination on the cardiac oxidative injury of CIS-injected rats

**Fig. 4:**
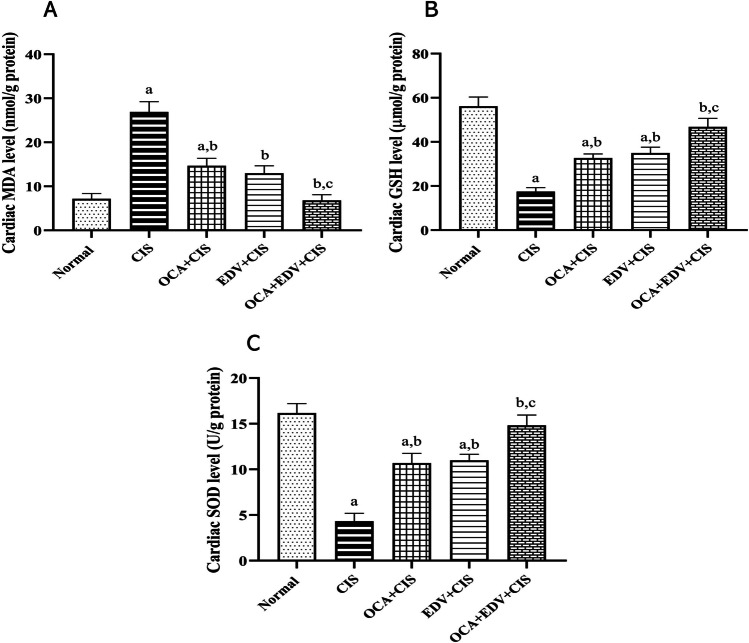
Effect of OCA or EDV and their combination on the cardiac oxidative injury of CIS-injected rats. **A** MDA, **B** GSH, and **C** SOD were measured spectrophotometrically. Results are represented as mean ± SE of 8 independent values and statistically conducted using a one-way ANOVA test at *P*-value < 0.05. a represents a significant difference from the normal control group. b represents a significant difference from the CIS control group. c represents a significant difference from the OCA + CIS group

Figure 4 illustrated a significant increase in cardiac MDA content and a significant decrease in cardiac antioxidants SOD and GSH levels following CIS injection. Conversely, OCA, EDV, and OCA plus EDV administration resulted in a marked decrease in MDA content, with a significant rise in SOD and GSH levels.

### Effect of OCA or EDV and their combination on cardiac Nrf2, PPARγ, and SIRT1 signals of CIS-intoxicated rats

**Fig. 5:**
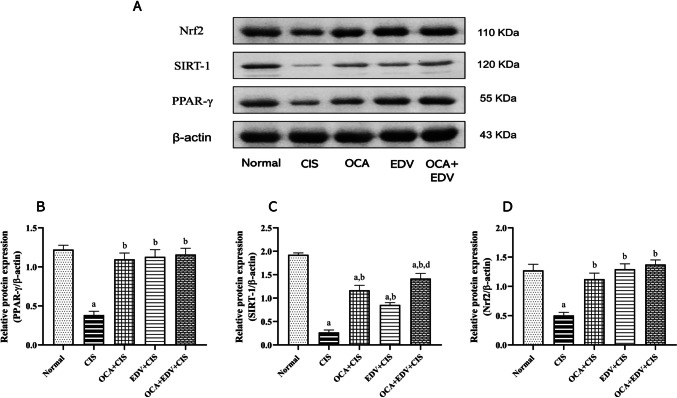
Effect of OCA or EDV and their combination on cardiac Nrf2, PPARγ, and SIRT1 signals of CIS-intoxicated rats. **A** Western blot bands of Nrf2, PPARγ, and SIRT1 protein expressions. **B** Semiquantitative analysis of PPARγ. **C** Semiquantitative analysis of SIRT1. **D** Semiquantitative analysis of Nrf2. Results are represented as mean ± SE of 3 independent values and statistically conducted using a one-way ANOVA test at *P*-value < 0.05. a represents a significant difference from the normal control group. b represents a significant difference from the CIS control group. d represents a significant difference from the EDV + CIS group

Western blotting was used to analyze the levels of protein expression for redox-sensitive signals Nrf2, PPAR, and SIRT1. Here, the proteins Nrf2, PPAR, and SIRT1 were all significantly downregulated in CIS-injected rats. In contrast, oral administration of OCA, EDV, and OCA plus EDV significantly upregulated Nrf2, PPARγ, and SIRT1 proteins (Fig. 5).

### Effect of OCA or EDV and their combination on cardiac inflammation of CIS-intoxicated rats

**Fig. 6:**
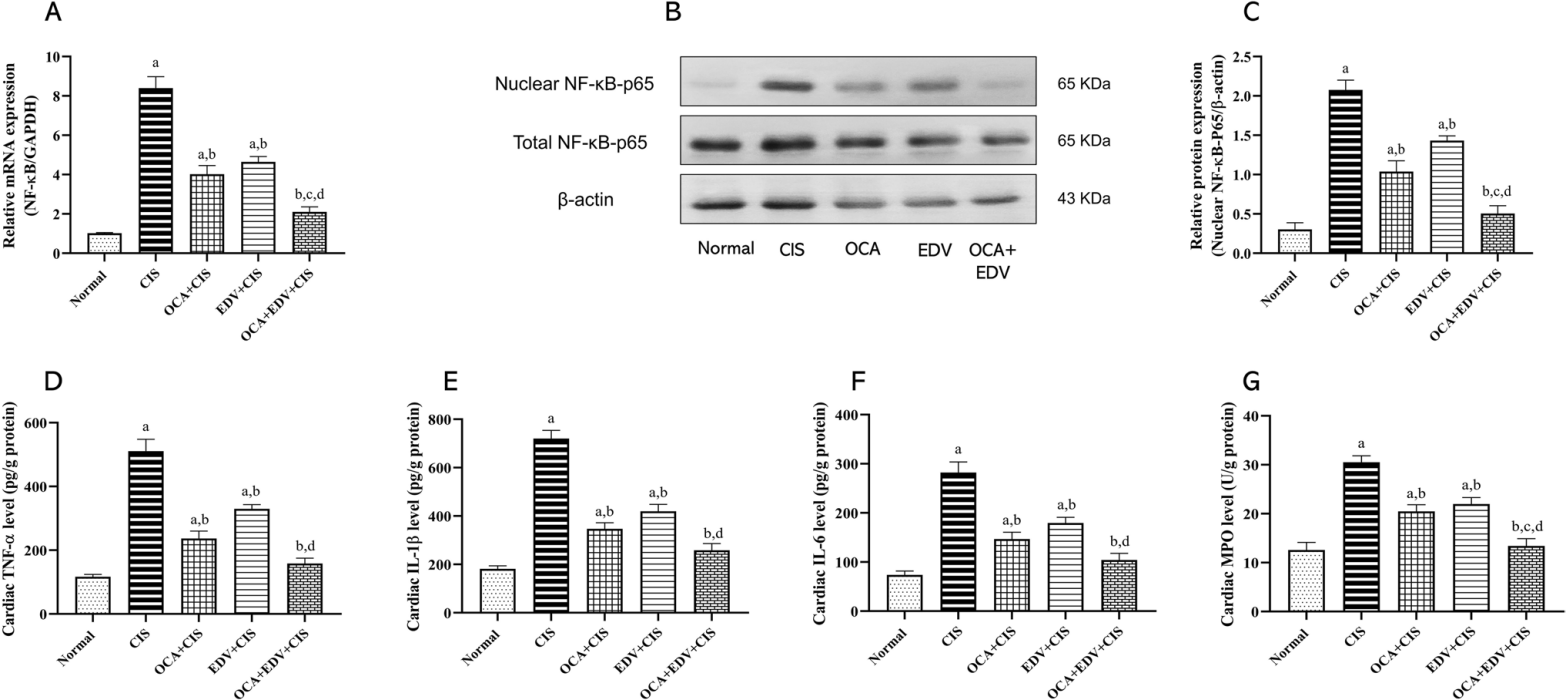
Effect of OCA or EDV and their combination on cardiac inflammation of CIS-challenged rats. **A** NF-κB mRNA expression, **B** Western blot bands for total and nuclear NF-κB-p65, **C** semiquantitative analysis of nuclear NF-κB-p65, **D** TNF-α, **E** IL-1β, **F** IL-6, **G** MPO. Results are represented as mean ± SE of 8 independent values and statistically conducted using a one-way ANOVA test at *P*-value < 0.05. a represents a significant difference from the normal control group. b represents a significant difference from the CIS control group. c represents a significant difference from the OCA + CIS group. d represents a significant difference from the EDV + CIS group

The injection of CIS markedly increased cardiac MPO enzymatic activity and cardiac cytokines TNF-α, IL-1β, and IL-6 levels, as well as NF-κB expression either mRNA or nuclear translocation, compared with normal control rats. Treatment with OCA, EDV, and OCA plus EDV showed a noticeable decline in cardiac TNF-α, IL-1β, IL-6, and NF-κB expressions compared with CIS-injected rats (Fig. 6).

### Effect of OCA or EDV and their combination on cardiac JAK1/STAT3 signals of CIS-intoxicated rats

**Fig. 7:**
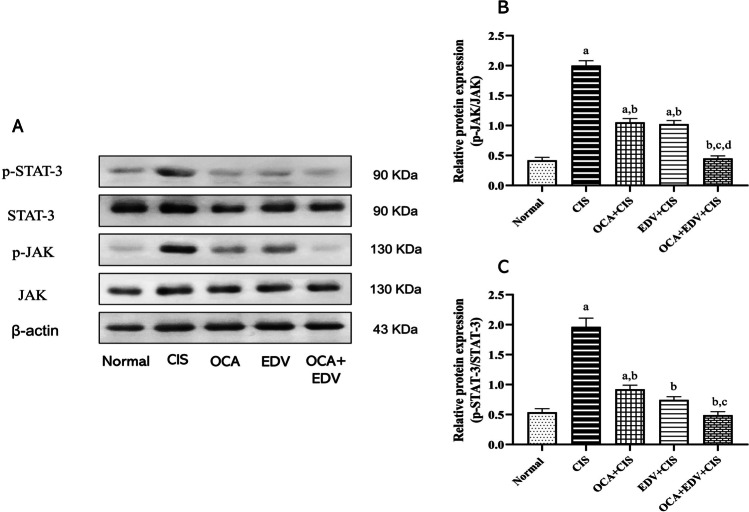
Effect of OCA or EDV and their combination on cardiac JAK1/STAT3 signals of CIS-challenged rats. **A** Western blot bands of JAK1, p-JAK1, STAT3, p-STAT3 protein expressions. **B** Semiquantitative analysis of p-JAK1/JAK. **C** Semiquantitative analysis of p-STAT3/STAT3. Results are represented as mean ± SE of 3 independent values and statistically conducted using a one-way ANOVA test at *P*-value < 0.05. a represents a significant difference from the normal control group. b represents a significant difference from the CIS control group. c represents a significant difference from the OCA + CIS group. d represents a significant difference from the EDV + CIS group

A western blotting assay assessed the protein expression of JAK1, p-JAK1, STAT3, and p-STAT3. As depicted in Fig. 7, when compared to normal rats, CIS injection increased JAK1 and STAT3 phosphorylation. On the other hand, oral administration of OCA, EDV, and their combination significantly suppressed JAK1 and STAT3 phosphorylation.

### Effect of OCA or EDV and their combination on cardiac p38MAPK/TLR4 and NLRP3 signals of CIS-intoxicated rats

**Fig. 8:**
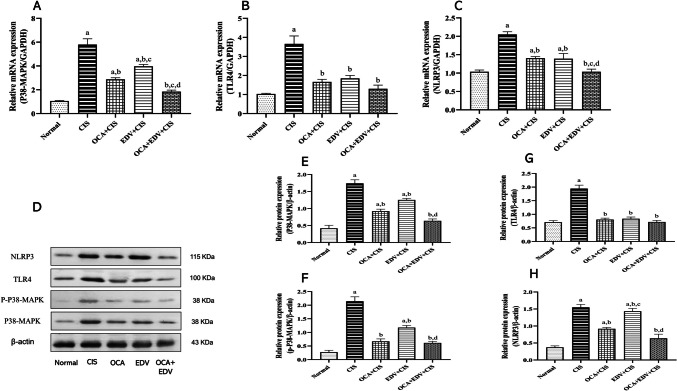
Effect of OCA or EDV and their combination on cardiac p38MAPK/TLR4 and NLRP3 signals of CIS-intoxicated rats. **A** p38MAPK; **B** TLR4; **C** NLRP3; **D** Western blot bands for p38MAPK, TLR4, and NLRP3; **E** semiquantitative analysis of p38MAPK; **F** semiquantitative analysis of p-p38MAPK; **G** semiquantitative analysis of TLR4; **H** semiquantitative analysis of NLRP3. Results are represented as mean ± SE of 8 independent values and statistically conducted using a one-way ANOVA test at *P*-value < 0.05. a represents a significant difference from the normal control group. b represents a significant difference from the CIS control group. c represents a significant difference from the OCA + CIS group. d represents a significant difference from the EDV + CIS group

The mRNA expression and protein levels of redox-sensitive signals p38MAPK, TLR4, and NLRP3 were assessed using a qRT-PCR assay, while the protein levels of these biomarkers were assessed by western blotting. In this regard, compared to normal rats, the heart of rats injected with CIS alone showed a significant upregulation of both mRNA and protein levels of p38MAPK, TLR4, and NLRP3. Conversely, compared to the CIS control group, oral administration of EDV, OCA, and their combination significantly downregulated p38MAPK, TLR4, and NLRP3 (Fig. 8). We emphasized our results by estimating phosphorylated p38MAPK expression. As expected, the protein expression of p-p38-MAPK was significantly upregulated in CIS control group as compared to normal control group. In contrast, oral administration of EDV, OCA, and their combination significantly reduced p38-MAPK phosphorylation as compared to CIS control group.

## Discussions

Despite its effectiveness in treating a wide range of cancers, the clinical use of CIS as a chemotherapy drug is limited because of the possibility of significant cardiac damage (Dugbartey et al. 2016; Herradón et al. 2017). We do not fully understand the exact mechanism underlying the cardiac damage induced by CIS. However, growing evidence shows ROS overproduction and inflammation are key contributors to CIS-induced cardiac injury (Miller et al. 2010; Barabas et al. 2008; El-Awady el et al. 2011; Qi et al. 2019; Qi et al. 2020). Consequently, it is crucial to thoroughly understand the underlying molecular mechanisms of CIS cardiac toxicity and identify new treatment targets.

In the current investigation, the administration of CIS led to a striking rise in serum levels of ALP, AST, CK-MB, LDH, and troponin-I, as well as significant cardiac histological abrasions. These data are in line with several previous studies (Bukhari et al. 2022; Xu et al. 2021; Gunturk et al. 2019). On the other hand, treatment with OCA, EDV, and their combinations indicated a significant cardiac protective effect, as seen by the decreased levels of these biomarkers. These effects of OCA and EDV can be related to their abilities to suppress oxidative stress and inflammation, preserving the structure and integrity of cardiac cells. Therefore, we aimed to examine the antioxidant and anti-inflammatory activities of OCA and EDV, pointing to the impact of Nrf2, PPARγ, SIRT1, NF-κB, JAK1/STAT3, and TLR4/p38MAPK signals in these effects.

In the present study, CIS injection increased TNF-α, IL-1β, and IL-6 cytokines and MPO enzymatic activity, indicating a severe inflammatory response. These data are in harmony with several investigations (Qi et al. 2020; Zhao et al. 2018; Xing et al. 2019). In contrast, administration of OCA, EDV, and their combination potently counteracted these elevations. The molecular mechanisms underlying the inflammatory damage of CIS-induced cardiotoxicity have received a lot of interest. In addition to their harmful effects on cellular macromolecules, ROS cause the production of inflammatory mediators such as inducible nitric oxide synthase, TNF-α, IL-1β, and IL-6 by activating TLR-4 and NF-κB (Baeuerle and Baichwal 1997; Asehnoune et al. 2004; Asami and Shimizu 2021). TLRs play a critical role in the innate immune system as pattern recognition receptors (Lundberg et al. 2013). Importantly, TLR4 mediates the inflammatory response in the heart (Chimenti et al. 2017; Lu et al. 2015). Recent studies have demonstrated that CIS activates TLR4 in different models, such as testicular (Hassanein et al. 2021), renal (Deng et al. 2020), and hepatic (Khedr et al. 2020) injuries. Numerous studies have shown that myocardial tissue injury is primarily caused by an inflammatory response and that this response is fostered by the TLR4 signal, which regulates the generation of proinflammatory mediators and promotes the NF-B pathway (Luo et al. 2020; Xiao et al. 2021). Previous studies reported that OCA and its derivatives had been shown to decrease the release of proinflammatory cytokines in osteoarthritis and acute liver failure (Guo et al. 2021; Pellicciari et al. 2002). TLR4 is involved in CIS-induced epithelium damage among the TLRs. TLR4 on renal parenchymal cells triggers several pathways, including MAPK, which increases the production of inflammatory cytokines and causes kidney damage (Miller et al. 2010).

Side by side, despite its significance in cell survival and proliferation, several data showed that NLRP3 activation has a key role CIS-induced toxicities (Jiang et al. 2021; Li et al. 2019). Moreover, strong evidence has been reported that NLRP3 activation is critical in myocardial inflammation and damage (Minutoli et al. 2016). NLRP3 inflammasome was activated by ROS, which was recently shown to have a significant role in promoting the inflammatory response (Liu et al. 2016). Additionally, the circulating proinflammatory cytokines activate the JAK/STAT system to start downstream signal transduction, and phosphorylated STAT proteins transform into potent transcription factors for particular STAT-target genes (Malemud and Pearlman 2009). JAK1 is necessary for receptor signaling by IL-6-type cytokines, even though these cytokines may activate other JAK kinase family members. The recruitment of inflammatory cells is encouraged by phosphorylated STAT3, intensifying the inflammatory response (Xin et al. 2020; Rawlings et al. 2004). In the current study, CIS injection elevated JAK1 and STAT3 phosphorylation. Conversely, oral administration of OCA, EDV, and their combination dramatically reduced JAK1 and STAT3 phosphorylation compared to the CIS control group. Taken together, OCA and EDV can attenuate TLR4/NF-κB/NLRP-3 and JAK1/STAT-3 pathway activation and, in turn, suppress inflammatory cytokines-induced myocardial damage.

The cardiomyocyte may suffer severe oxidative damage from excessive ROS, which could damage the cells and cause cardiac damage (Deavall et al. 2012; Costa et al. 2013). Interestingly, several studies showed that antioxidants or ROS scavengers might significantly reduce the cardiac damage induced by CIS (Yüce et al. 2007; Qi et al. 2022). Oxidative stress caused by CIS was evidenced by increased MDA and decreased GSH and SOD. The generation of reactive oxygen species (ROS) is increased by CIS, and this might affect cellular macromolecules by, for example, peroxidizing membrane lipids and thereby altering the membrane’s fluidity and permeability. Furthermore, ROS’s high oxidative activity may lead to the depletion of GSH and antioxidant enzymes. These findings are supported by several previous studies (Yüce et al. 2007; Qi et al. 2022). In contrast, OCA, EDV, and their combinations markedly decreased heart MDA content with a marked boosting of the antioxidant status by increasing cardiac antioxidants GSH and SOD. In various experimental models, EDV reduced oxidative stress. As evidenced by the improvement in heart function and the decrease in histological injury, treatment with EDV had an antioxidant protective effect on the heart (Hassanein et al. 2020a; Hassan et al. 2015). In diabetic cardiomyopathy, EDV improved cardiac function by upregulating Nrf2 and SIRT1 and potently counteracted heart oxidative injury (Ji et al. 2016). Also, OCA exhibited antioxidant effects in different models, such as valproic acid-induced hepatotoxicity (Gai et al. 2020) and hepatorenal syndrome in ascitic cirrhotic rats (Tsai et al. 2020).

To emphasize the mechanism(s) behind the protective effect of OCA and EDV against CIS-induced oxidative and inflammatory responses in the heart, we evaluated the changes in Nrf2, PPAR, and SIRT1 signals. Nrf2 can directly inhibit NF-B and the inflammatory response while concurrently activating anti-inflammatory pathways to limit the inflammatory cascade (Wardyn et al. 2015). Previous investigations have shown that Nrf2 activation reduces the harmful effects of chemotherapy on the heart (Wang et al. 2022; Hassanein et al. 2020a). In parallel, PPARγ upregulates the expression of antioxidant genes (Okuno et al. 2010) and inhibits NF-κB activation (Mateu et al. 2015). In various models of cardiac damage, including diabetic cardiomyopathy (Gbr et al. 2021) and sepsis-induced cardiac dysfunction (Peng et al. 2017), the anti-inflammatory impact of PPAR has been mediated by suppressing NF-κB. Also, SIRT1 ensures cell survival by deacetylating substrate proteins under oxidative stress (Farghali et al. 2019; Raynes et al. 2013) and negative regulation of NF-κB activity (Chen et al. 2005). In the present investigation, CIS injection decreased Nrf2, PPARγ, and SIRT1. In contrast, OCA and EDV increased Nrf2, PPARγ, and SIRT1. Consequently, improvements in antioxidant defenses and reduced oxidative stress and inflammation were attributed to Nrf2, PPARγ, and SIRT1 activation in the heart of CIS-administered rats treated with OCA and EDV.

## Conclusions

The results of the present study revealed that CIS cardiotoxicity could be prevented by using EDV, OCA, and combination therapy. This effect could be attributed to the interplay of antioxidant and anti-inflammatory capabilities of tested agents through modulation of Nrf2/PPARγ/SIRT1, JAK1/STAT3/NF-κB, and TLR4/P38MAPK signaling pathways. Notably, the combination therapy between OCA and EDV exhibited the highest protective outcomes rather than each drug alone. Their effects on tumor reduction and cytotoxic activity of CIS should be considered in future work. In this regard, the promising results of this combination attract to recommend use in the regimen of CIS therapy.

### Supplementary Information

Below is the link to the electronic supplementary material.

Supplementary file1 (PDF 541 KB)

## Data Availability

The datasets used and/or analyzed during the current study are available from the corresponding authors upon reasonable request.

## References

[CR1] Abenavoli L, Procopio AC, Fagoonee S, Pellicano R, Carbone M, Luzza F, Invernizzi P (2020) Primary biliary cholangitis and bile acid farnesoid X receptor agonists. Diseases (Basel, Switzerland) 8 (2). 10.3390/diseases8020020 10.3390/diseases8020020 PMC7348889 32532037

[CR2] Al-Kahtani MA, Abdel-Moneim AM, Elmenshawy OM, El-Kersh MA (2014) Hemin attenuates cisplatin-induced acute renal injury in male rats. Oxid Med Cell Longev 2014:476430. 10.1155/2014/476430 25332751 10.1155/2014/476430 PMC4190123

[CR3] Asami J, Shimizu T (2021) Structural and functional understanding of the toll-like receptors. Protein Sci 30(4):761–772. 10.1002/pro.4043 33576548 10.1002/pro.4043 PMC7980524

[CR4] Asehnoune K, Strassheim D, Mitra S, Kim JY, Abraham E (2004) Involvement of reactive oxygen species in Toll-like receptor 4-dependent activation of NF-kappa B. J Immunol (Baltimore, Md: 1950) 172(4):2522–2529. 10.4049/jimmunol.172.4.2522 10.4049/jimmunol.172.4.2522 14764725

[CR5] Baeuerle PA, Baichwal VR (1997) NF-kappa B as a frequent target for immunosuppressive and anti-inflammatory molecules. Adv Immunol 65:111–137 9238509 10.1016/S0065-2776(08)60742-7

[CR6] Barabas K, Milner R, Lurie D, Adin C (2008) Cisplatin: a review of toxicities and therapeutic applications. Vet Comp Oncol 6(1):1–18. 10.1111/j.1476-5829.2007.00142.x 19178659 10.1111/j.1476-5829.2007.00142.x

[CR7] Bradford MM (1976) A rapid and sensitive method for the quantitation of microgram quantities of protein utilizing the principle of protein-dye binding. Anal Biochem 72:248–254 942051 10.1016/0003-2697(76)90527-3

[CR8] Bukhari IA, Mohamed OY, Alhowikan AM, Lateef R, Hagar H, Assiri RA, Alqahtani WMA (2022) Protective effect of rutin trihydrate against dose-dependent, cisplatin-induced cardiac toxicity in isolated perfused rat’s heart. Cureus 14(1):e21572. 10.7759/cureus.21572 35228931 10.7759/cureus.21572 PMC8866754

[CR9] Chen J, Zhou Y, Mueller-Steiner S, Chen LF, Kwon H, Yi S, Mucke L, Gan L (2005) SIRT1 protects against microglia-dependent amyloid-beta toxicity through inhibiting NF-kappaB signaling. J Biol Chem 280(48):40364–40374. 10.1074/jbc.M509329200 16183991 10.1074/jbc.M509329200

[CR10] Chimenti C, Verardo R, Scopelliti F, Grande C, Petrosillo N, Piselli P, De Paulis R, Frustaci A (2017) Myocardial expression of Toll-like receptor 4 predicts the response to immunosuppressive therapy in patients with virus-negative chronic inflammatory cardiomyopathy. Eur J Heart Fail 19(7):915–925. 10.1002/ejhf.796 28370906 10.1002/ejhf.796

[CR11] Costa VM, Carvalho F, Duarte JA, Bastos MdL, Remião F (2013) The heart as a target for xenobiotic toxicity: the cardiac susceptibility to oxidative stress. Chem Res Toxicol 26(9):1285–1311 23902227 10.1021/tx400130v

[CR12] Dasari S, Tchounwou PB (2014) Cisplatin in cancer therapy: molecular mechanisms of action. Eur J Pharmacol 740:364–378. 10.1016/j.ejphar.2014.07.025 25058905 10.1016/j.ejphar.2014.07.025 PMC4146684

[CR13] Deavall DG, Martin EA, Horner JM, Roberts R (2012) Drug-induced oxidative stress and toxicity. J Toxicol 2012:645460. 10.1155/2012/645460 10.1155/2012/645460 PMC3420138 22919381

[CR14] Deng JS, Jiang WP, Chen CC, Lee LY, Li PY, Huang WC, Liao JC, Chen HY, Huang SS, Huang GJ (2020) Cordyceps cicadae mycelia ameliorate cisplatin-induced acute kidney injury by suppressing the TLR4/NF-κB/MAPK and activating the HO-1/Nrf2 and Sirt-1/AMPK pathways in mice. Oxid Med Cell Longev 2020:7912763. 10.1155/2020/7912763 32089779 10.1155/2020/7912763 PMC7026739

[CR15] Dou W, Zhang J, Sun A, Zhang E, Ding L, Mukherjee S, Wei X, Chou G, Wang ZT, Mani S (2013) Protective effect of naringenin against experimental colitis via suppression of Toll-like receptor 4/NF-κB signalling. Br J Nutr 110(4):599–608. 10.1017/s0007114512005594 23506745 10.1017/s0007114512005594 PMC3726555

[CR16] Dugbartey GJ, Peppone LJ, de Graaf IA (2016) An integrative view of cisplatin-induced renal and cardiac toxicities: molecular mechanisms, current treatment challenges and potential protective measures. Toxicology 371:58–66. 10.1016/j.tox.2016.10.001 27717837 10.1016/j.tox.2016.10.001 PMC5586594

[CR17] el El-Awady SE, Moustafa YM, Abo-Elmatty DM, Radwan A (2011) Cisplatin-induced cardiotoxicity: mechanisms and cardioprotective strategies. Eur J Pharmacol 650(1):335–341. 10.1016/j.ejphar.2010.09.085 21034734 10.1016/j.ejphar.2010.09.085

[CR18] El-Shoura EAM, Messiha BAS, Sharkawi SMZ, Hemeida RAM (2018) Perindopril ameliorates lipopolysaccharide-induced brain injury through modulation of angiotensin-II/angiotensin-1-7 and related signaling pathways. Eur J Pharmacol 834:305–317. 10.1016/j.ejphar.2018.07.046 30059682 10.1016/j.ejphar.2018.07.046

[CR19] Emekli-Alturfan E, Alev B, Tunali S, Oktay S, Tunali-Akbay T, Ozturk LK, Yanardag R, Yarat A (2015) Effects of edaravone on cardiac damage in valproic acid induced toxicity. Ann Clin Lab Sci 45(2):166–172 25887870

[CR20] Farghali H, Kemelo MK, Canova NK (2019) SIRT1 modulators in experimentally induced liver injury. Oxid Med Cell Longev 2019:8765954. 10.1155/2019/8765954 31281594 10.1155/2019/8765954 PMC6589266

[CR21] Feldman AT, Wolfe D (2014) Tissue processing and hematoxylin and eosin staining. Methods Mol Biol 1180:31–43. 10.1007/978-1-4939-1050-2_3 25015141 10.1007/978-1-4939-1050-2_3

[CR22] Ferrigno A, Palladini G, Di Pasqua LG, Berardo C, Richelmi P, Cadamuro M, Fabris L, Perlini S, Adorini L, Vairetti M (2020) Obeticholic acid reduces biliary and hepatic matrix metalloproteinases activity in rat hepatic ischemia/reperfusion injury. PLoS ONE 15(9):e0238543. 10.1371/journal.pone.0238543 32911524 10.1371/journal.pone.0238543 PMC7482919

[CR23] Gai Z, Krajnc E, Samodelov SL, Visentin M, Kullak-Ublick GA (2020) Obeticholic acid ameliorates valproic acid-induced hepatic steatosis and oxidative stress. Mol Pharmacol 97(5):314–323. 10.1124/mol.119.118646 32098797 10.1124/mol.119.118646

[CR24] Gbr AA, Abdel Baky NA, Mohamed EA, Zaky HS (2021) Cardioprotective effect of pioglitazone and curcumin against diabetic cardiomyopathy in type 1 diabetes mellitus: impact on CaMKII/NF-κB/TGF-β1 and PPAR-γ signaling pathway. Naunyn Schmiedebergs Arch Pharmacol 394(2):349–360. 10.1007/s00210-020-01979-y 32984914 10.1007/s00210-020-01979-y

[CR25] Ghosh S (2019) Cisplatin: the first metal based anticancer drug. Bioorg Chem 88:102925. 10.1016/j.bioorg.2019.102925 31003078 10.1016/j.bioorg.2019.102925

[CR26] Gunturk EE, Yucel B, Gunturk I, Yazici C, Yay A, Kose K (2019) The effects of N-acetylcysteine on cisplatin induced cardiotoxicity. Bratisl Lek Listy 120(6):423–428. 10.4149/bll_2019_068 31223022 10.4149/bll_2019_068

[CR27] Guo D, He L, Gao Y, Jin C, Lin H, Zhang L, Wang L, Zhou Y, Yao J, Duan Y, Yang R, Qiu W, Jiang W (2021) Obeticholic acid derivative, T-2054 suppresses osteoarthritis via inhibiting NF-κB-signaling pathway. Int J Mol Sci 22 (8). 10.3390/ijms22083807 10.3390/ijms22083807 PMC8067620 33916928

[CR28] Hassan MQ, Akhtar MS, Akhtar M, Ali J, Haque SE, Najmi AK (2015) Edaravone protects rats against oxidative stress and apoptosis in experimentally induced myocardial infarction: biochemical and ultrastructural evidence. Redox Rep 20(6):275–281. 10.1179/1351000215y.0000000011 25893851 10.1179/1351000215y.0000000011 PMC6837640

[CR29] Hassanein EHM, Abd El-Ghafar OAM, Ahmed MA, Sayed AM, Gad-Elrab WM, Ajarem JS, Allam AA, Mahmoud AM (2020a) Edaravone and acetovanillone upregulate Nrf2 and PI3K/Akt/mTOR signaling and prevent cyclophosphamide cardiotoxicity in rats. Drug Des Dev Ther 14:5275–5288. 10.2147/dddt.S281854 10.2147/dddt.S281854 PMC7721127 33299300

[CR30] Hassanein EHM, Sayed AM, Hussein OE, Mahmoud AM (2020b) Coumarins as modulators of the Keap1/Nrf2/ARE signaling pathway. Oxid Med Cell Longev 2020:1675957. 10.1155/2020/1675957 32377290 10.1155/2020/1675957 PMC7196981

[CR31] Hassanein EHM, Abdel-Wahab BA, Ali FEM, Abd El-Ghafar OAM, Kozman MR, Sharkawi SMZ (2021) Trans-ferulic acid ameliorates cisplatin-induced testicular damage via suppression of TLR4, P38-MAPK, and ERK1/2 signaling pathways. Environ Sci Pollut Res Int 28(31):41948–41964. 10.1007/s11356-021-13544-y 33792844 10.1007/s11356-021-13544-y

[CR32] Herradón E, González C, Uranga JA, Abalo R, Martín MI, López-Miranda V (2017) Characterization of cardiovascular alterations induced by different chronic cisplatin treatments. Front Pharmacol 8:196. 10.3389/fphar.2017.00196 28533750 10.3389/fphar.2017.00196 PMC5420557

[CR33] Iguchi T, Nishikawa M, Chang B, Muroya O, Sato EF, Nakatani T, Inoue M (2004) Edaravone inhibits acute renal injury and cyst formation in cisplatin-treated rat kidney. Free Radical Res 38(4):333–341. 10.1080/10715760310001646886 15190930 10.1080/10715760310001646886

[CR34] Ivanova EA, Parolari A, Myasoedova V, Melnichenko AA, Bobryshev YV, Orekhov AN (2015) Peroxisome proliferator-activated receptor (PPAR) gamma in cardiovascular disorders and cardiovascular surgery. J Cardiol 66(4):271–278. 10.1016/j.jjcc.2015.05.004 26072262 10.1016/j.jjcc.2015.05.004

[CR35] Ji L, Liu Y, Zhang Y, Chang W, Gong J, Wei S, Li X, Qin L (2016) The antioxidant edaravone prevents cardiac dysfunction by suppressing oxidative stress in type 1 diabetic rats and in high-glucose-induced injured H9c2 cardiomyoblasts. Can J Physiol Pharmacol 94(9):996–1006. 10.1139/cjpp-2015-0587 27376621 10.1139/cjpp-2015-0587

[CR36] Jiang S, Zhang H, Li X, Yi B, Huang L, Hu Z, Li A, Du J, Li Y, Zhang W (2021) Vitamin D/VDR attenuate cisplatin-induced AKI by down-regulating NLRP3/caspase-1/GSDMD pyroptosis pathway. J Steroid Biochem Mol Biol 206:105789. 10.1016/j.jsbmb.2020.105789 33259938 10.1016/j.jsbmb.2020.105789

[CR37] Kalogeropoulos AP, Georgiopoulou VV, Butler J (2012) From risk factors to structural heart disease: the role of inflammation. Heart Fail Clin 8(1):113–123. 10.1016/j.hfc.2011.08.002 22108731 10.1016/j.hfc.2011.08.002

[CR38] Khedr LH, Rahmo RM, Farag DB, Schaalan MF, El Magdoub HM (2020) Crocin attenuates cisplatin-induced hepatotoxicity via TLR4/NF-κBp50 signaling and BAMBI modulation of TGF-β activity: Involvement of miRNA-9 and miRNA-29. Food Chem Toxicol 140:111307. 10.1016/j.fct.2020.111307 32259551 10.1016/j.fct.2020.111307

[CR39] Li S, Lin Q, Shao X, Mou S, Gu L, Wang L, Zhang Z, Shen J, Zhou Y, Qi C, Jin H, Pang H, Ni Z (2019) NLRP3 inflammasome inhibition attenuates cisplatin-induced renal fibrosis by decreasing oxidative stress and inflammation. Exp Cell Res 383(1):111488. 10.1016/j.yexcr.2019.07.001 31276670 10.1016/j.yexcr.2019.07.001

[CR40] Li C, Yang J, Wang Y, Qi Y, Yang W, Li Y (2020) Farnesoid X receptor agonists as therapeutic target for cardiometabolic diseases. Front Pharmacol 11:1247. 10.3389/fphar.2020.01247 32982723 10.3389/fphar.2020.01247 PMC7479173

[CR41] Liu X, Zhang Z, Ruan J, Pan Y, Magupalli VG, Wu H, Lieberman J (2016) Inflammasome-activated gasdermin D causes pyroptosis by forming membrane pores. Nature 535(7610):153–158. 10.1038/nature18629 27383986 10.1038/nature18629 PMC5539988

[CR42] Livak KJ, Schmittgen TD (2001) Analysis of relative gene expression data using real-time quantitative PCR and the 2− ΔΔCT method. Methods 25(4):402–408 11846609 10.1006/meth.2001.1262

[CR43] Loboda A, Damulewicz M, Pyza E, Jozkowicz A, Dulak J (2016) Role of Nrf2/HO-1 system in development, oxidative stress response and diseases: an evolutionarily conserved mechanism. Cell Mol Life Sci 73(17):3221–3247. 10.1007/s00018-016-2223-0 27100828 10.1007/s00018-016-2223-0 PMC4967105

[CR44] Lu M, Tang F, Zhang J, Luan A, Mei M, Xu C, Zhang S, Wang H, Maslov LN (2015) Astragaloside IV attenuates injury caused by myocardial ischemia/reperfusion in rats via regulation of toll-like receptor 4/nuclear factor-κB signaling pathway. Phytother Res 29(4):599–606. 10.1002/ptr.5297 25604645 10.1002/ptr.5297

[CR45] Lundberg AM, Ketelhuth DF, Johansson ME, Gerdes N, Liu S, Yamamoto M, Akira S, Hansson GK (2013) Toll-like receptor 3 and 4 signalling through the TRIF and TRAM adaptors in haematopoietic cells promotes atherosclerosis. Cardiovasc Res 99(2):364–373. 10.1093/cvr/cvt033 23417039 10.1093/cvr/cvt033

[CR46] Luo M, Yan D, Sun Q, Tao J, Xu L, Sun H, Zhao H (2020) Ginsenoside Rg1 attenuates cardiomyocyte apoptosis and inflammation via the TLR4/NF-kB/NLRP3 pathway. J Cell Biochem 121(4):2994–3004. 10.1002/jcb.29556 31709615 10.1002/jcb.29556

[CR47] Malemud CJ, Pearlman E (2009) Targeting JAK/STAT signaling pathway in inflammatory diseases. Curr Signal Transduct Ther 4(3):201–221. 10.2174/157436209789057467 10.2174/157436209789057467

[CR48] Manktelow A, Meyer AA (1986) Lack of correlation between decreased chemotaxis and susceptibility to infection in burned rats. J Trauma 26(2):143–148. 10.1097/00005373-198602000-00008 3944838 10.1097/00005373-198602000-00008

[CR49] Marklund SL (1985) Superoxide dismutase isoenzymes in tissues and plasma from New Zealand black mice, nude mice and normal BALB/c mice. Mutat Res 148(1–2):129–134. 10.1016/0027-5107(85)90216-7 3969077 10.1016/0027-5107(85)90216-7

[CR50] Mateu A, Ramudo L, Manso MA, De Dios I (2015) Cross-talk between TLR4 and PPARgamma pathways in the arachidonic acid-induced inflammatory response in pancreatic acini. Int J Biochem Cell Biol 69:132–141. 10.1016/j.biocel.2015.10.022. (**S1357-2725(15)30047-9 [pii]**) 26510582 10.1016/j.biocel.2015.10.022

[CR51] Mihara M, Uchiyama M (1978) Determination of malonaldehyde precursor in tissues by thiobarbituric acid test. Anal Biochem 86(1):271–278. 10.1016/0003-2697(78)90342-1 655387 10.1016/0003-2697(78)90342-1

[CR52] Miller RP, Tadagavadi RK, Ramesh G, Reeves WB (2010) Mechanisms of cisplatin nephrotoxicity. Toxins (Basel) 2(11):2490–2518. 10.3390/toxins2112490 22069563 10.3390/toxins2112490 PMC3153174

[CR53] Minutoli L, Puzzolo D, Rinaldi M, Irrera N, Marini H, Arcoraci V, Bitto A, Crea G, Pisani A, Squadrito F, Trichilo V, Bruschetta D, Micali A, Altavilla D (2016) ROS-mediated NLRP3 inflammasome activation in brain, heart, kidney, and testis ischemia/reperfusion injury. Oxid Med Cell Longev 2016:2183026. 10.1155/2016/2183026 27127546 10.1155/2016/2183026 PMC4835650

[CR54] Molh AK, Ting LC, Khan J, Al-Jashamy K, Jaafar H, Islam MN (2008) Histopathological studies of cardiac lesions after an acute high dose administration of methamphetamine. Malays J Med Sci 15(1):23–30 22589611 PMC3341894

[CR55] Moresco EM, LaVine D, Beutler B (2011) Toll-like receptors. Curr Biol 21(13):R488-493. 10.1016/j.cub.2011.05.039 21741580 10.1016/j.cub.2011.05.039

[CR56] Okuno Y, Matsuda M, Miyata Y, Fukuhara A, Komuro R, Shimabukuro M, Shimomura I (2010) Human catalase gene is regulated by peroxisome proliferator activated receptor-gamma through a response element distinct from that of mouse. Endocr J 57(4):303–309 20075562 10.1507/endocrj.K09E-113

[CR57] Packer M (2020) Cardioprotective effects of sirtuin-1 and its downstream effectors: potential role in mediating the heart failure benefits of SGLT2 (sodium-glucose cotransporter 2) inhibitors. Circ Heart Fail 13(9):e007197. 10.1161/circheartfailure.120.007197 32894987 10.1161/circheartfailure.120.007197

[CR58] Pellicciari R, Fiorucci S, Camaioni E, Clerici C, Costantino G, Maloney PR, Morelli A, Parks DJ, Willson TM (2002) 6alpha-ethyl-chenodeoxycholic acid (6-ECDCA), a potent and selective FXR agonist endowed with anticholestatic activity. J Med Chem 45(17):3569–3572. 10.1021/jm025529g 12166927 10.1021/jm025529g

[CR59] Peng S, Xu J, Ruan W, Li S, Xiao F (2017) PPAR-γ activation prevents septic cardiac dysfunction via inhibition of apoptosis and necroptosis. Oxid Med Cell Longev 2017:8326749. 10.1155/2017/8326749 28845215 10.1155/2017/8326749 PMC5560091

[CR60] Qi L, Luo Q, Zhang Y, Jia F, Zhao Y, Wang F (2019) Advances in toxicological research of the anticancer drug cisplatin. Chem Res Toxicol 32(8):1469–1486. 10.1021/acs.chemrestox.9b00204 31353895 10.1021/acs.chemrestox.9b00204

[CR61] Qi Y, Ying Y, Zou J, Fang Q, Yuan X, Cao Y, Cai Y, Fu S (2020) Kaempferol attenuated cisplatin-induced cardiac injury via inhibiting STING/NF-κB-mediated inflammation. Am J Transl Res 12(12):8007–8018 33437376 PMC7791507

[CR62] Qi Y, Fu S, Pei D, Fang Q, Xin W, Yuan X, Cao Y, Shu Q, Mi X, Luo F (2022) Luteolin attenuated cisplatin-induced cardiac dysfunction and oxidative stress via modulation of Keap1/Nrf2 signaling pathway. Free Radical Res 56(2):209–221. 10.1080/10715762.2022.2067042 35468014 10.1080/10715762.2022.2067042

[CR63] Rawlings JS, Rosler KM, Harrison DA (2004) The JAK/STAT signaling pathway. J Cell Sci 117(8):1281–1283. 10.1242/jcs.00963 15020666 10.1242/jcs.00963

[CR64] Raynes R, Brunquell J, Westerheide SD (2013) Stress inducibility of SIRT1 and its role in cytoprotection and cancer. Genes Cancer 4(3–4):172–182. 10.1177/1947601913484497 24020008 10.1177/1947601913484497 PMC3764474

[CR65] Rothstein JD (2017) Edaravone: a new drug approved for ALS. Cell 171(4):725 29100067 10.1016/j.cell.2017.10.011

[CR66] Sedlak J, Lindsay RH (1968) Estimation of total, protein-bound, and nonprotein sulfhydryl groups in tissue with Ellman’s reagent. Anal Biochem 25(1):192–205. 10.1016/0003-2697(68)90092-4 4973948 10.1016/0003-2697(68)90092-4

[CR67] Sherif IO (2021) Hepatoprotective effect of arjunolic acid against cisplatin-induced hepatotoxicity: targeting oxidative stress, inflammation, and apoptosis. J Biochem Mol Toxicol 35(4):e22714. 10.1002/jbt.22714 33491850 10.1002/jbt.22714

[CR68] Singh V, Ubaid S (2020) Role of silent information regulator 1 (SIRT1) in regulating oxidative stress and inflammation. Inflammation 43(5):1589–1598. 10.1007/s10753-020-01242-9 32410071 10.1007/s10753-020-01242-9

[CR69] Towbin H, Staehelin T, Gordon J (1979) Electrophoretic transfer of proteins from polyacrylamide gels to nitrocellulose sheets: procedure and some applications. Proc Natl Acad Sci USA 76(9):4350–4354. 10.1073/pnas.76.9.4350 388439 10.1073/pnas.76.9.4350 PMC411572

[CR70] Tsai YL, Liu CW, Hsu CF, Huang CC, Lin MW, Huang SF, Li TH, Lee KC, Hsieh YC, Yang YY, Lee TY, Liu HM, Huang YH, Hou MC, Lin HC (2020) Obeticholic acid ameliorates hepatorenal syndrome in ascitic cirrhotic rats by down-regulating the renal 8-iso-PGF2α-activated COX-TXA2 pathway. Clin Sci (Lond) 134(15):2055–2073. 10.1042/cs20200452 32725149 10.1042/cs20200452

[CR71] Wang SH, Tsai KL, Chou WC, Cheng HC, Huang YT, Ou HC, Chang YC (2022) Quercetin mitigates cisplatin-induced oxidative damage and apoptosis in cardiomyocytes through Nrf2/HO-1 signaling pathway. Am J Chin Med 50(5):1281–1298. 10.1142/s0192415x22500537 35670059 10.1142/s0192415x22500537

[CR72] Wardyn JD, Ponsford AH, Sanderson CM (2015) Dissecting molecular cross-talk between Nrf2 and NF-κB response pathways. Biochem Soc Trans 43(4):621–626 26551702 10.1042/BST20150014 PMC4613495

[CR73] Watanabe T, Tahara M, Todo S (2008) The novel antioxidant edaravone: from bench to bedside. Cardiovasc Ther 26(2):101–114. 10.1111/j.1527-3466.2008.00041.x 18485133 10.1111/j.1527-3466.2008.00041.x

[CR74] Xiao Z, Kong B, Fang J, Qin T, Dai C, Shuai W, Huang H (2021) Ferrostatin-1 alleviates lipopolysaccharide-induced cardiac dysfunction. Bioengineered 12(2):9367–9376. 10.1080/21655979.2021.2001913 34787054 10.1080/21655979.2021.2001913 PMC8809987

[CR75] Xin P, Xu X, Deng C, Liu S, Wang Y, Zhou X, Ma H, Wei D, Sun S (2020) The role of JAK/STAT signaling pathway and its inhibitors in diseases. Int Immunopharmacol 80:106210. 10.1016/j.intimp.2020.106210 31972425 10.1016/j.intimp.2020.106210

[CR76] Xing JJ, Hou JG, Liu Y, Zhang RB, Jiang S, Ren S, Wang YP, Shen Q, Li W, Li XD, Wang Z (2019) Supplementation of saponins from leaves of Panax quinquefolius mitigates cisplatin-evoked cardiotoxicity via inhibiting oxidative stress-associated inflammation and apoptosis in mice. Antioxidants (Basel) 8(9). 10.3390/antiox8090347 10.3390/antiox8090347 PMC6769973 31480577

[CR77] Xu J, Zhang B, Chu Z, Jiang F, Han J (2021) Wogonin alleviates cisplatin-induced cardiotoxicity in mice via inhibiting gasdermin D-mediated pyroptosis. J Cardiovasc Pharmacol 78(4):597–603. 10.1097/fjc.0000000000001085 34651602 10.1097/fjc.0000000000001085 PMC8492184

[CR78] Yüce A, Ateşşahin A, Ceribaşi AO, Aksakal M (2007) Ellagic acid prevents cisplatin-induced oxidative stress in liver and heart tissue of rats. Basic Clin Pharmacol Toxicol 101(5):345–349. 10.1111/j.1742-7843.2007.00129.x 17910619 10.1111/j.1742-7843.2007.00129.x

[CR79] Zhang XH, Matsuda N, Jesmin S, Sakuraya F, Gando S, Kemmotsu O, Hattori Y (2003) Normalization by edaravone, a free radical scavenger, of irradiation-reduced endothelial nitric oxide synthase expression. Eur J Pharmacol 476(1–2):131–137. 10.1016/s0014-2999(03)02151-4 12969758 10.1016/s0014-2999(03)02151-4

[CR80] Zhao L, Xing C, Sun W, Hou G, Yang G, Yuan L (2018) Lactobacillus supplementation prevents cisplatin-induced cardiotoxicity possibly by inflammation inhibition. Cancer Chemother Pharmacol 82(6):999–1008. 10.1007/s00280-018-3691-8 30276453 10.1007/s00280-018-3691-8

