# Supplementary material for: Edaravone and obeticholic acid protect against cisplatin-induced heart toxicity by suppressing oxidative stress and inflammation and modulating Nrf2, TLR4/p38MAPK, and JAK1/STAT3/NF-κB signals
Source: Naunyn Schmiedebergs Arch Pharmacol. 2024 Jan 29;397(8):5649–62. doi: 10.1007/s00210-024-02956-5 (PMC11329704; doi:10.1007/s00210-024-02956-5)
Supplement: Supplementary file 1 — Supplementary file1 (PDF 541 KB) [file 210_2024_2956_MOESM1_ESM.pdf]

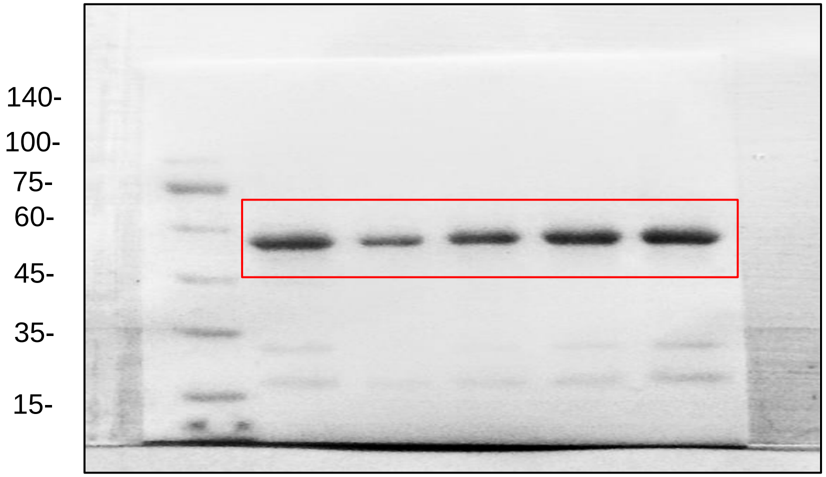


PPAR-γ

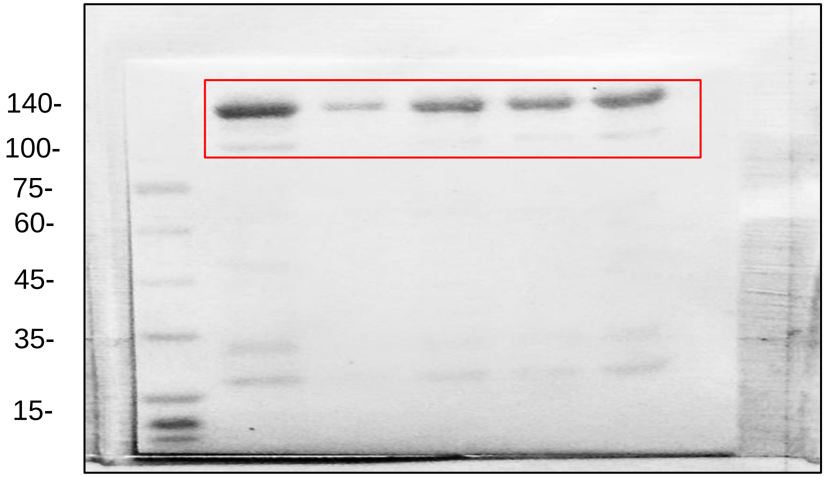


SIRT-1

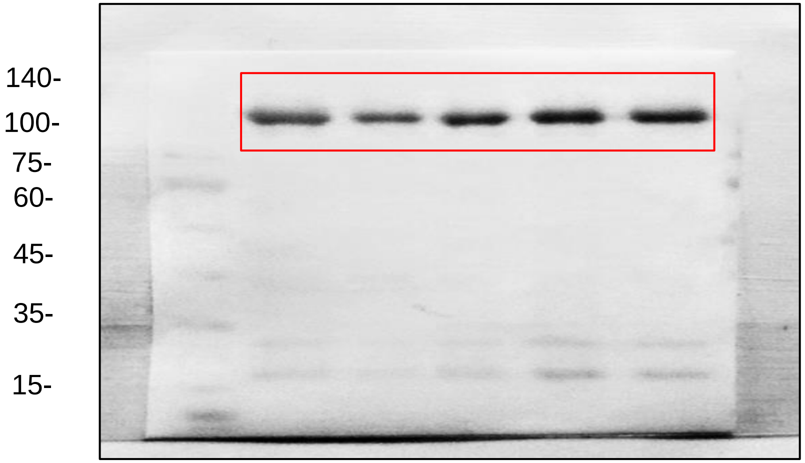


Nrf2

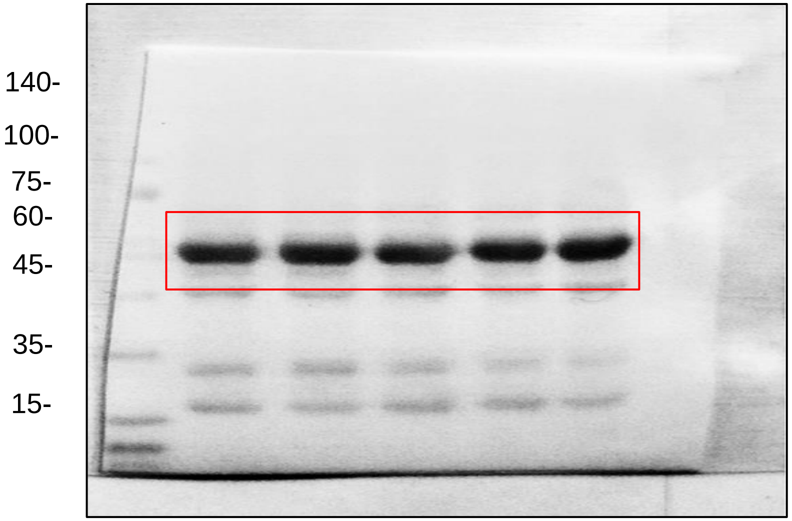


β-actin

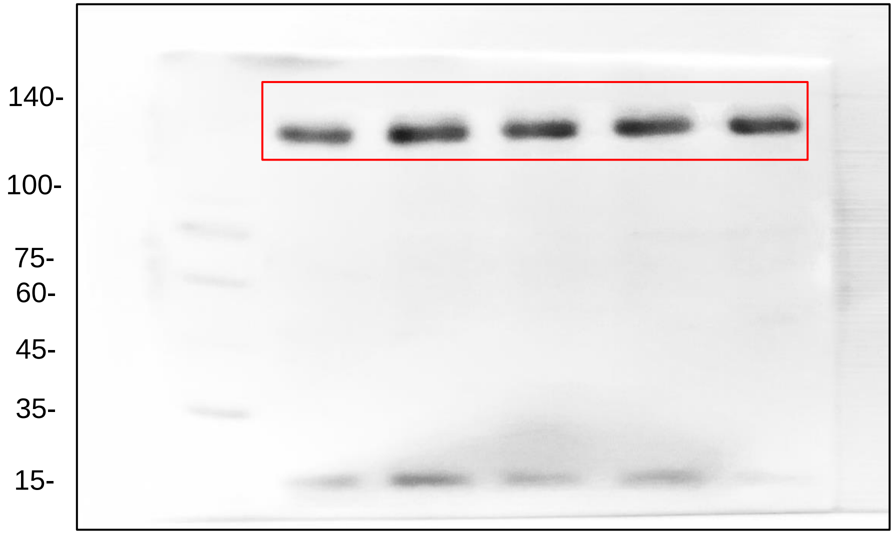


JAK

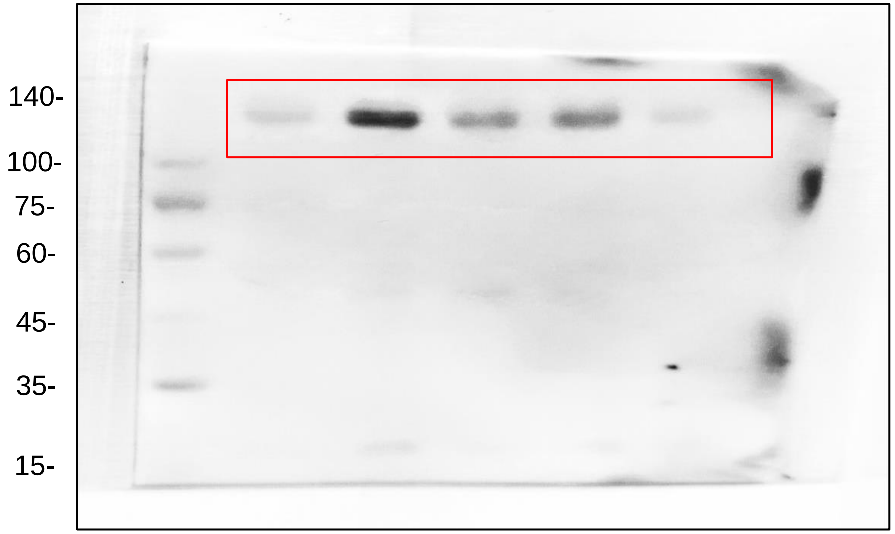


p-JAK

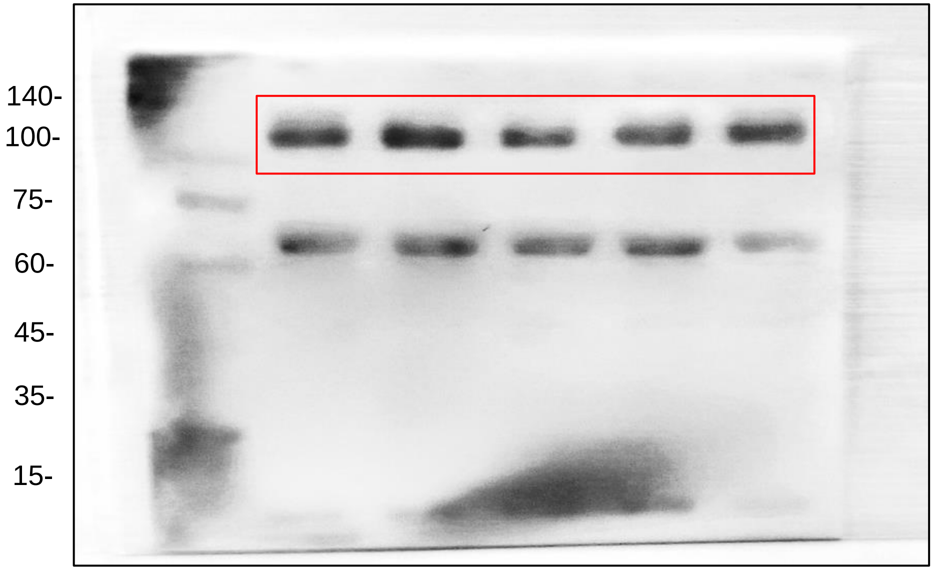


STAT-3

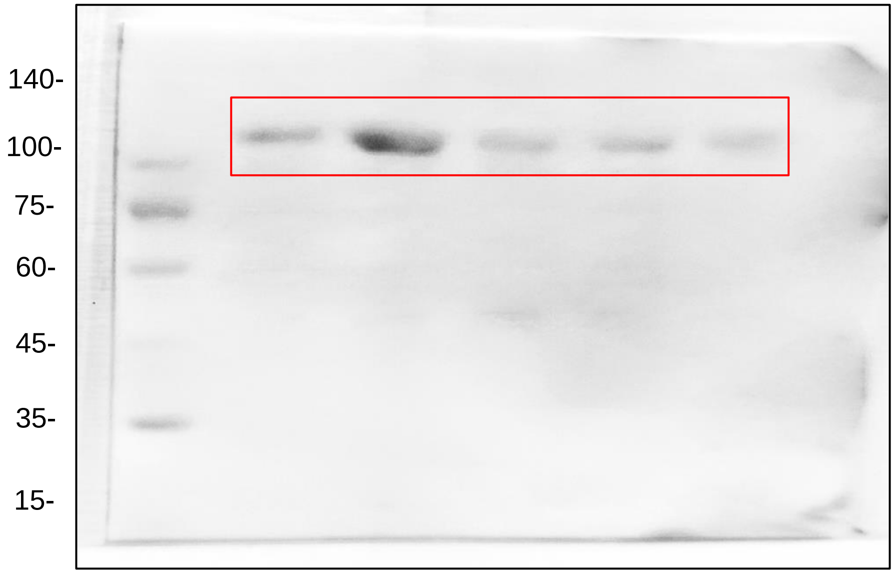


p-STAT-3

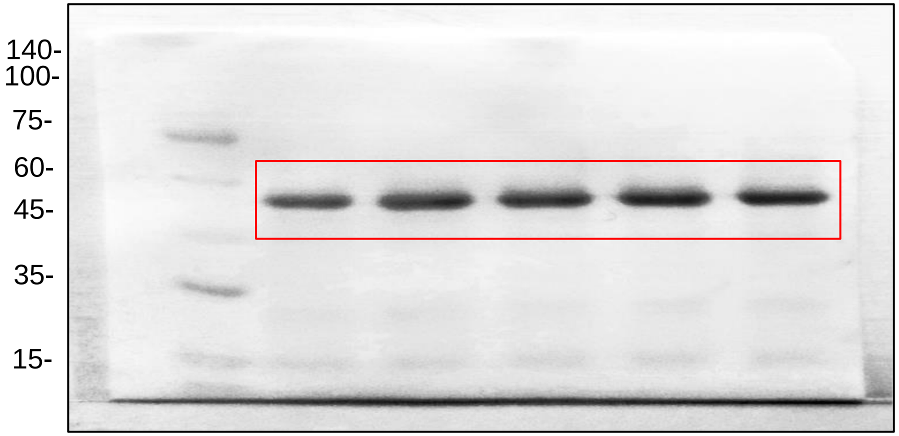


β-actin

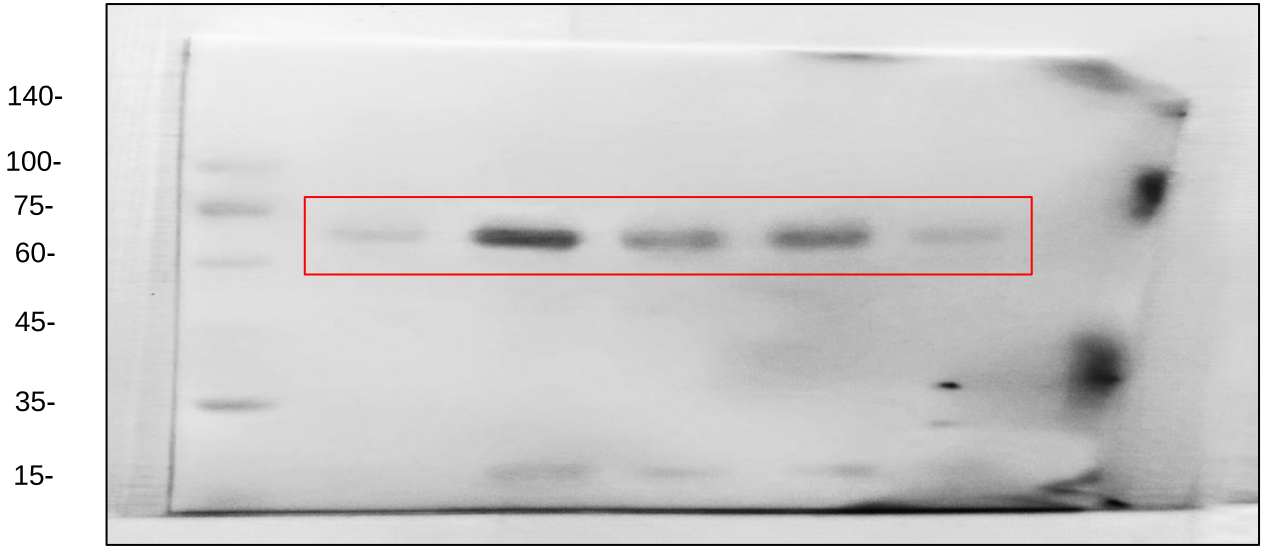


Nuclear NF-κB-p65

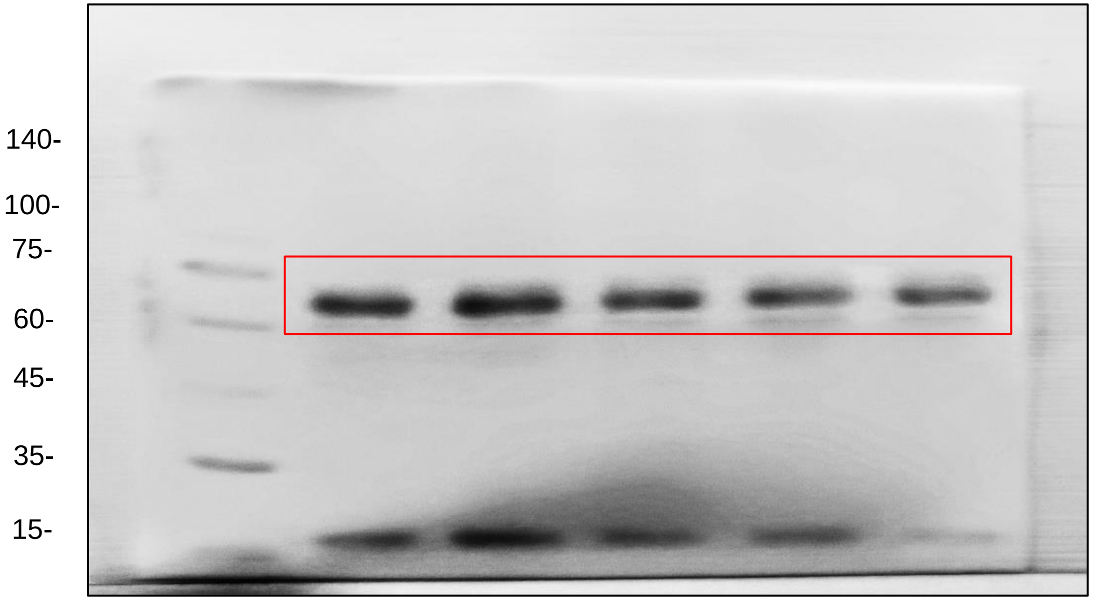


Total NF-κB-p65

140-
100-
75-
60-
45-
35-
15-

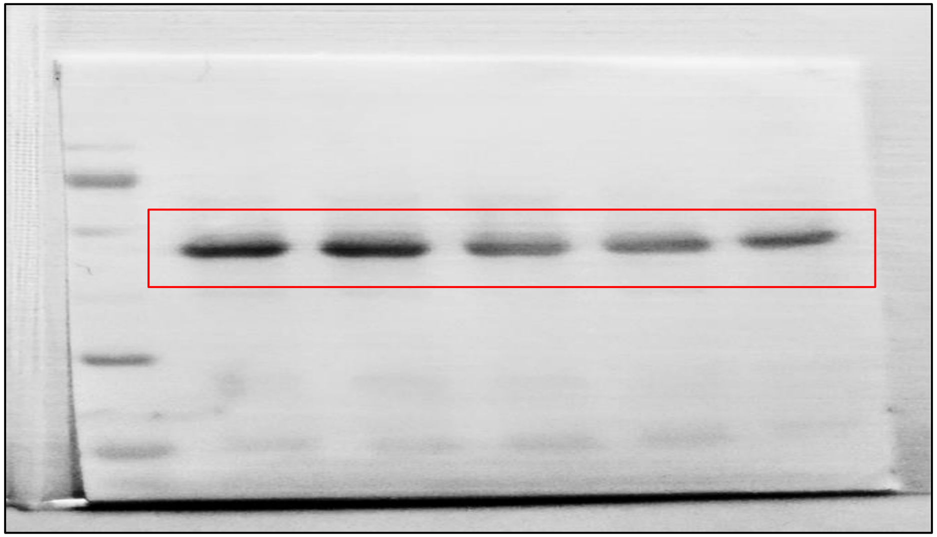

β-actin

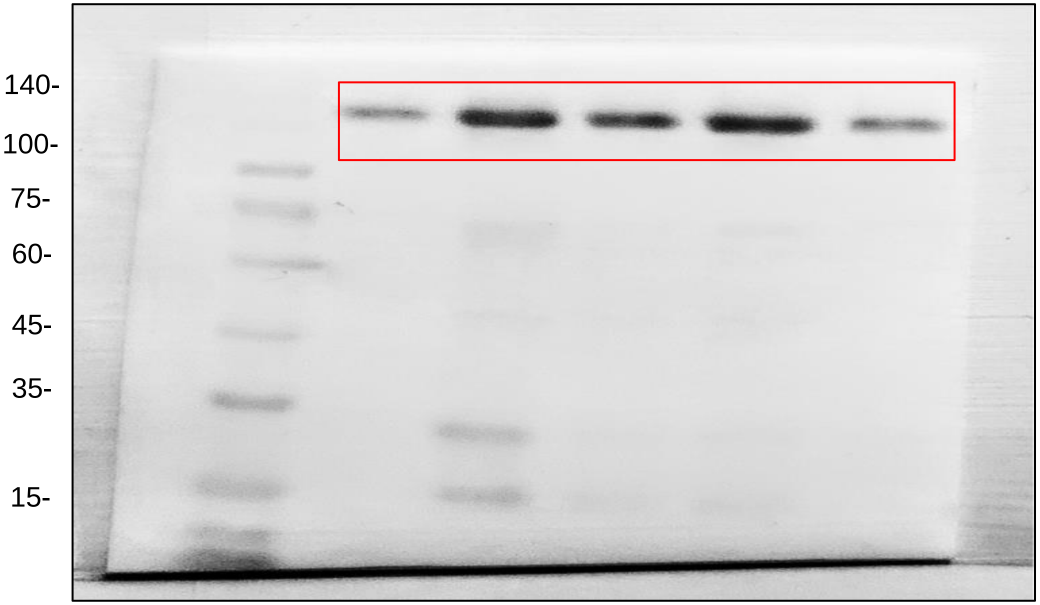


NLRP3

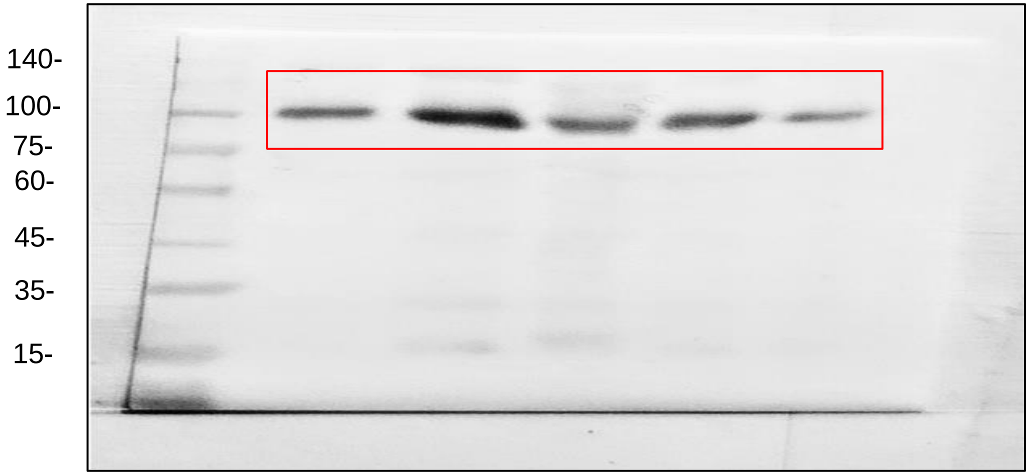


TLR4

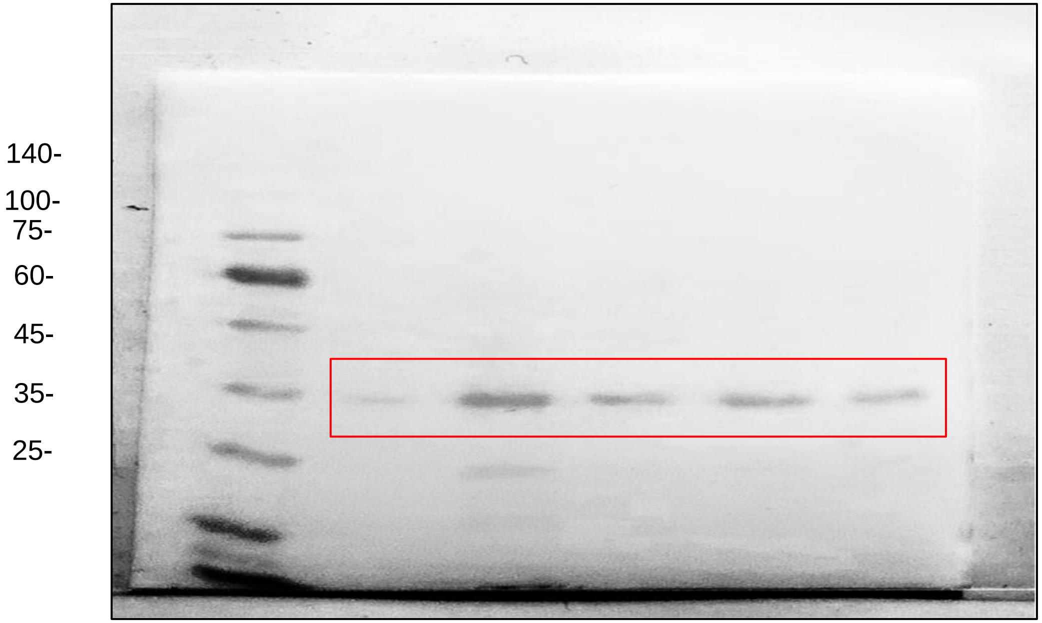


p-P38-MAPK

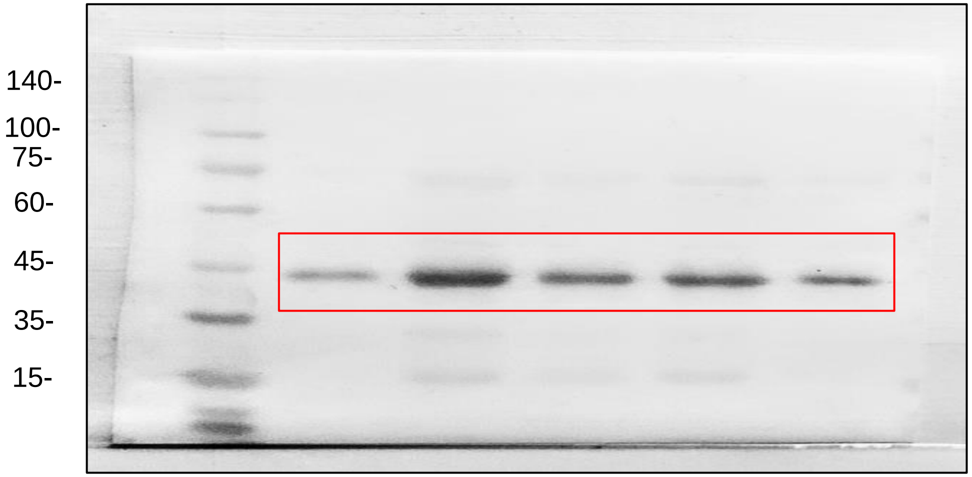


P38-MAPK

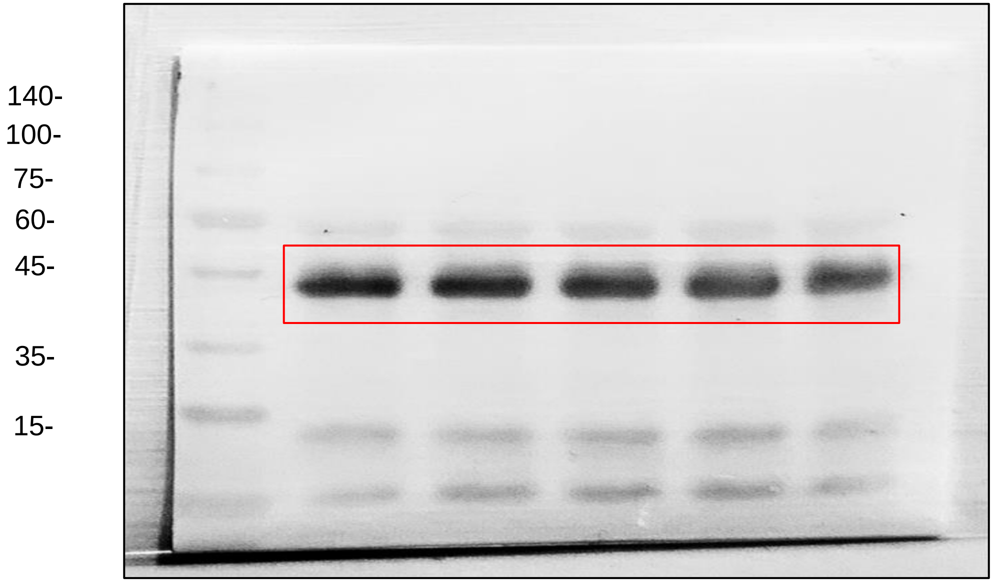


β-actin
